# Dual Release of Antibiotics with Additive Effect from Cleavable Hydrogels Based on Amino/Thiol–Yne Click Chemistry

**DOI:** 10.1021/acs.biomac.6c01230

**Published:** 2026-08-17

**Authors:** Sara Bescós-Ramo, Cristina Yus, Manuel Arruebo, Milagros Piñol, Luis Oriol

**Affiliations:** † Instituto de Nanociencia y Materiales de Aragón (INMA), CSIC-Universidad de Zaragoza, 50009 Zaragoza, Spain; ‡ Departamento de Química Orgánica, Facultad de Ciencias, Universidad de Zaragoza, 50009 Zaragoza, Spain; § Department of Chemical Engineering and Environmental Technology, Universidad de Zaragoza, 50018 Zaragoza, Spain; ∥ Aragon Health Research Institute (IIS Aragón), 50009 Zaragoza, Spain

## Abstract

Dual-antibiotic loaded poly­(ethylene glycol) (PEG)-based hydrogels were developed as antimicrobial wound dressings combining covalent and physical antibiotic incorporation. Hydrogels were prepared through amino-yne click conjugation of gentamicin sulfate (Gs) and thiol-yne cross-linking, followed by ciprofloxacin (CPX) loading via swelling-diffusion. The materials exhibited tunable gelation time, swelling capacity, and drug release profiles depending on thiol content. A dual-release mechanism was achieved, consisting of an initial burst release of CPX followed by a sustained, pH-responsive release of conjugated Gs through acid cleavage of β-aminoacrylate linkages. The hydrogels showed effective wide spectrum antibacterial activity against methicillin-resistant *Staphylococcus aureus* and *Pseudomonas aeruginosa*, while cytotoxicity studies confirmed cytocompatibility at bactericidal concentrations. The results support the potential of these hydrogels as safe and effective dual-antibiotic wound dressings, allowing for the immediate elimination of invading bacteria after wounding (burst release), while maintaining long-term antibiotic release to minimize reinfection risk.

## Graphical abstract

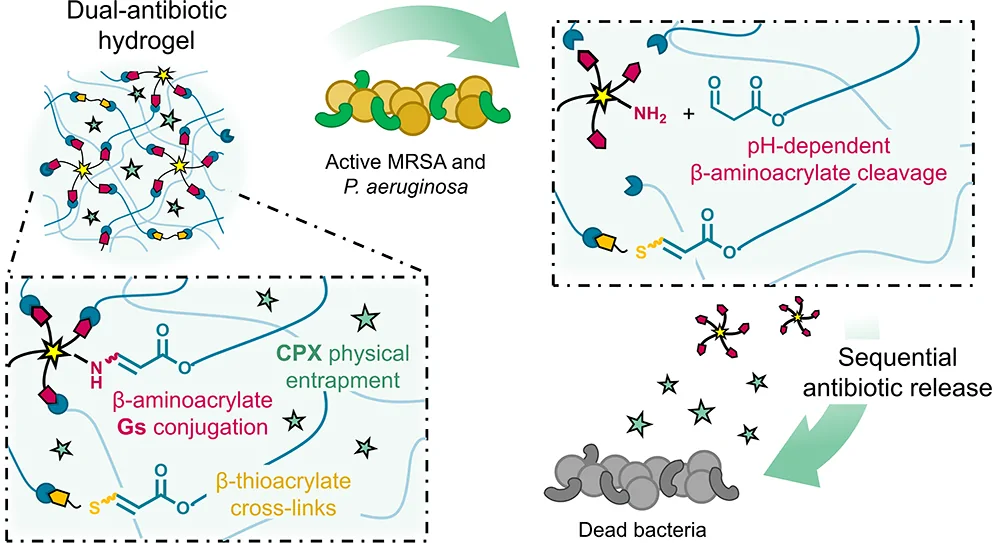

## 1 Introduction

Bacterial infections represent a major complication in wound healing, often delaying or impairing the normal physiological repair process. Failure to restore skin structure and function within a time frame, normally sufficient for tissue repair, results in the development of chronic wounds, which impose a substantial burden on patients and healthcare systems.^1^ Accordingly, promoting rapid and effective healing remains a key objective in skin tissue engineering.^2^ Hydrogels have emerged as promising wound dressings due to their high water content, which maintains a moist environment and supports cell proliferation, as well as their high exudate absorption capacity, gas exchange ability, and tunable physicochemical properties.^3^ In addition, they can serve as carriers for antibacterial agents, enabling localized drug delivery.^4^ Antibiotics, although reserved for severe cases in which bone is compromised, remain essential for treating wound infections,^5^ selectively targeting bacteria while showing limited toxicity toward mammalian cells at therapeutic doses. However, their systemic administration is associated with adverse effects and contributes to the development of resistance, motivating controlled local delivery strategies. Moreover, the growing prevalence of resistant strains often limits the efficacy of monotherapy, prompting alternative approaches such as combination therapy.^6–8^ The use of multiple antibiotics can reduce required doses, broaden antibacterial coverage, and mitigate resistance development, although outcomes depend on drug interactions and the target microorganisms.

Strategies to incorporate two drugs within a single delivery system have been widely explored to enhance therapeutic efficacy.^9^ In the simplest approach, both agents are physically encapsulated, leading to similar diffusion-controlled release profiles typically limited to hours or up to 1 day.^10,11^ However, effective wound healing often requires differentiated release kinetics, combining an initial burst to eradicate accidental bacteria present after wounding with sustained release to prevent reinfection.^12^ To address this, alternative strategies based on distinct incorporation modes have been developed, including the covalent conjugation of one therapeutic agent to enable prolonged and controlled release upon bond cleavage.^13^ When such linkages are stimuli-responsive, for instance to acidic pH, drug release can be further accelerated under the conditions characteristic of infected wounds in early stages.^14^ Common pH-sensitive linkers include hydrazones, oximes, acetals/ketals, and imines,^15^ the latter being widely used for amine-containing antibiotics via Schiff base formation and subsequent acid-triggered hydrolysis.^16–19^

Amino-yne click chemistry, involving the addition of amines to electron-deficient alkynes, has emerged as an efficient strategy for both the synthesis of polymeric systems and bioconjugation, as it proceeds spontaneously in the absence of catalysts under ambient conditions and in aqueous media, enabling mild and versatile network formation.^20–23^ Beyond its synthetic advantages, this reaction generates β-aminoacrylate linkages that are cleavable under acidic conditions (pH < 5.5), enabling the recovery of the initial amine.^24^ In the context of hydrogels, Langer et al. pioneered the preparation of poly­(ethylene glycol) (PEG)-based networks via this catalyst-free amino-yne click reaction,^25^ while Huang and Jiang demonstrated pH-induced sol–gel transitions in chitosan/PEG systems.^26^ Building on these advances, amino-yne hydrogels have also been explored for wound healing applications. For instance, Deng et al. reported a multifunctional hydrogel for the treatment of infected diabetic wounds,^27^ and Zhang et al. developed PEG-based hydrogels incorporating basic fibroblast growth factor and the antibiotic gentamicin sulfate (Gs, *i.e.*, an aminoglycoside antibiotic) via noncovalent interactions.^28^ However, in these systems, antibiotic incorporation relied on physical encapsulation, resulting in diffusion-controlled release, as evidenced by the complete release of Gs within 12 h.

Recently, we reported amino-yne-based PEG tetra-alkynoate hydrogels, in which the overall degradation rate can be tuned by varying pH, temperature, polymer concentration, or the amine used as a linker.^29^ Moreover, we demonstrated the recovery of the conjugated amine upon β-aminoacrylate cleavage, with distinct release kinetics at acidic and physiological pH. Notably, this system was limited to model amines and did not address drug conjugation. Inspired by these findings, we envisioned that amino-yne click chemistry could be extended to the covalent conjugation of amino-containing drugs, enabling controlled release from hydrogel networks for wound healing applications. Indeed, this approach has been demonstrated in nanoparticle-based systems, where doxorubicin (*i.e.*, a chemotherapeutic drug) is covalently linked via β-aminoacrylate linkages, with minimal release at physiological pH and accelerated release under acidic conditions.^24,30^

Accordingly, in this work, hydrogel network formation was achieved using a PEG tetra-alkynoate macromolecule (denoted as PEGalk) via spontaneous amino-yne and thiol-yne click reactions conducted under ambient conditions, involving the amine groups of the antibiotic Gs and the thiol groups of dithiothreitol (DTT) (Figure 1a, PEGT_*x*_-Gs hydrogel). Gs was selected for covalent conjugation due to its five amino groups, enabling its dual role as a therapeutic agent and multifunctional cross-linker, as well as its high solubility at physiological pH (7.4), which facilitates amino-yne reaction in aqueous media. Importantly, previous studies have shown that cross-linkers rich in primary amines, such as Gs, lead to relatively fast hydrogel degradation.^29^ Therefore, the nucleophilic thiol-yne reaction, a highly efficient chemistry widely used for the formation of robust hydrogel materials,^31,32^ was incorporated into the formulation as a fast and spontaneous strategy to introduce β-thioacrylate linkages, with the expectation of a slower response to acidic environments compared to β-aminoacrylates and thereby potentially delaying hydrogel degradation and Gs release. In order to enhance antibacterial efficacy, combination therapy was also explored by physically incorporating ciprofloxacin (CPX, *i.e.*, a fluoroquinolone antibiotic) as a second antibiotic (Figure 1b, PEGT_*x*_-GsCPX hydrogel). Although both are broad-spectrum antibiotics, their modes of action differ significantly, enabling them to target selected bacterial strains through different mechanisms. Whereas Gs involves binding to the bacterial 30S ribosomal subunit, therefore interfering with protein synthesis,^33^ CPX targets bacterial DNA replication by inhibiting bacterial DNA gyrase and topoisomerase IV.^34^ Moreover, Gs and CPX have previously been used in combination, reporting additive or synergistic antimicrobial effects depending on their relative concentrations and the targeted bacterial strain.^35^ Synergistic activity has been reported in MRSA isolates resistant to Gs but sensitive to CPX when both antibiotics were administered together.^36^ In Gram-negative bacteria, additive or synergistic effects have also been observed against planktonic cultures and biofilms of *Pseudomonas aeruginosa* (*P. aeruginosa*) and *Escherichia coli* (*E. coli*), although the outcome remains strain-dependent.^37–39^ They have also been coloaded into electrospun gelatin fibers, resulting in a significant reduction in bacterial load in an *in vivo* murine model of a deep burn infected with *P. aeruginosa*.^40^ Importantly, their different modes of encapsulation are expected to enable distinct release kinetics for each therapeutic agent, combining rapid diffusion-driven release of CPX, to remove the initial bacterial contamination, with the slower cleavage of covalent bonds of Gs, thereby helping to prevent reinfection during the healing. In addition, the acid-induced cleavage of the β-aminoacrylate linkages might allow tuning the release kinetics according to the wound microenvironment, enabling the dual-antibiotic delivery system to exhibit pH-dependent release behavior (Figure 1c) and supporting prolonged infection control. Herein, the bactericidal activity of the as-prepared materials was evaluated *in vitro* against planktonic methicillin-resistant *Staphylococcus aureus* (MRSA) and *P. aeruginosa*. Their cytotoxicity was assessed at the tested doses using two model eukaryotic cell lines, fibroblasts and macrophages.

**Figure 1:**
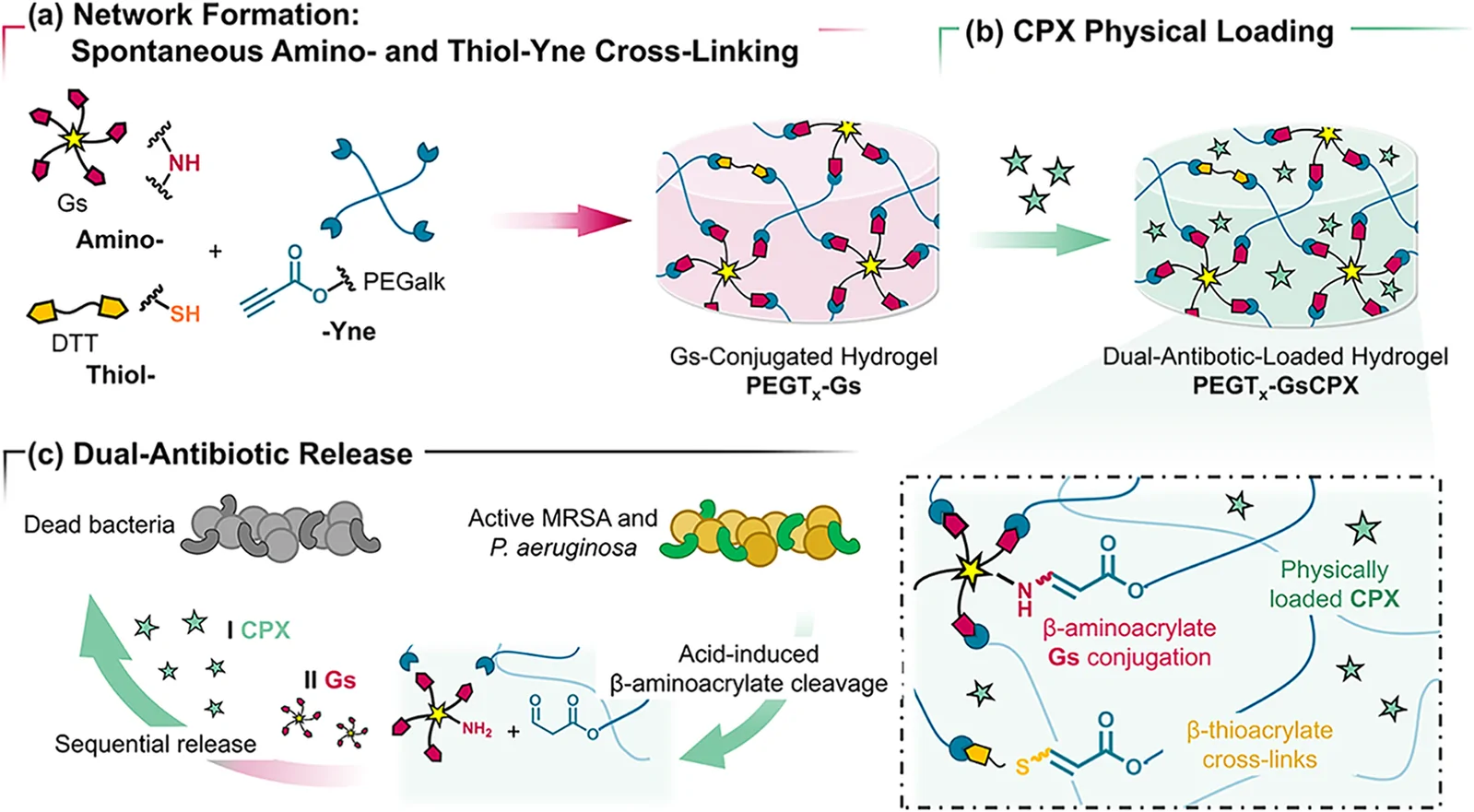
Overview of the two-step preparation of dual-antibiotic hydrogels, including (a) network formation through spontaneous amino-yne and thiol–yne click chemistries with Gs conjugation, (b) physical loading of CPX, and (c) dual-antibiotic release.

## 2 Experimental (Materials and Methods)

### 2.1 Materials

PEGalk was synthesized according to a previously reported procedure,^29^ using four-arm PEG (pentaerythritol core, *M*_n_ = 10 000 g/mol) as starting material. Gs and DTT were purchased from Sigma-Aldrich. CPX hydrochloride was purchased from BLD Pharmatech GmbH (Germany). Dulbecco’s Phosphate-Buffered Saline (DPBS), Dulbecco’s modified Eagle’s medium (DMEM w/stable glutamine) and antibiotic-antimycotic solution (penicillin-streptomycin-amphotericin B) were purchased from Biowest (France). Tryptic soy broth (TSB) was purchased from Condalab (Spain). Tryptic soy agar (TSA) plates were prepared using Chromocult dehydrated media produced by Merck KGaA (Germany). Fetal bovine serum (FBS) was purchased from Gibco Waltham (US). Blue Cell Viability Assay kit was purchased from Abnova (Taiwan). Clinical MRSA USA300 strain was kindly provided by Dr. Cristina Prat (Institut d’Investigació en Ciènces de la Salut Germans Trias i Pujol, Badalona, Spain). *P. aeruginosa* ATCC10145 was purchased from Ielab (Spain). J774A.1 mouse monocyte-macrophages cell line (ATCC-TIB-67TM) was purchased from LGC Standards (Barcelona, Spain). Human dermal fibroblasts (NHDF-Ad) were sourced from Lonza (Basel, Switzerland). Phosphate buffer (PB, 0.1 M, pH = 7.4) was prepared by mixing sodium phosphate dibasic heptahydrate (72 mM) and sodium phosphate monobasic monohydrate (28 mM) in Milli-Q water. Acetate buffer (0.1 M, pH = 5.0) was prepared by mixing sodium acetate (70 mM) and acetic acid (30 mM) in Milli-Q water.

### 2.2 Preparation of Hydrogels

#### 2.2.1 PEGT_*x*_-Gs Hydrogel Preparation

Hydrogels were prepared in 1.5 mL plastic vials (8 mm × 35 mm) with a final mass of 100 mg (PEGalk + solvent), assuming the pH = 7.4 phosphate buffer (PB) density is approximately 1 g/mL. First, 20 mg of PEGalk was weighed into the plastic vial to achieve a polymer concentration of 20 wt % in the final mass. Then, it was dissolved in 40 μL of PB (50% of final PB volume) by orbital stirring for 4 h at room temperature. Gs solution in PB was prepared so that a 1:1 molar ratio of alkynoate:amino group was reached after adding Gs in 20 μL of PB (25% of the final PB volume). After 10 s of vortex mixing, solutions were allowed to react at a controlled temperature of 25 °C for 1 h. Then, DTT solution in PB was prepared so that 1:0.15, 1:0.25 or 1:0.35 molar ratios of alkynoate:thiol group were reached after adding the cross-linker in 20 μL of PB (25% of the final PB volume). After 10 s of vortex mixing, hydrogels were allowed to set at 25 °C for 18 h. The resulting hydrogels were denoted as PEGT_*x*_-Gs, where *x* represents the mol % of thiol groups relative to the alkynoate groups used during network formation. Compilation of mass, volume and concentration of reagents used for the preparation of the different hydrogels is presented in Table S1 (Supporting Information). For comparison, PEGT_*x*_ hydrogels were prepared following an analogous procedure but without Gs (Supporting Information, Table S2).

#### 2.2.2 PEGT_*x*_-GsCPX Hydrogel Preparation (CPX Loading)

CPX was loaded into the Gs-conjugated hydrogels (PEGalkT_*x*_-Gs) using a swelling-diffusion method. Due to the low solubility of CPX at physiological pH, the PB present within the hydrogels was removed prior to drug loading to prevent CPX precipitation. This was achieved by immersing the hydrogels in Milli-Q water (3 mL) for 4 h at 25 °C, with the aqueous medium being refreshed every hour. The hydrogels were then lyophilized overnight. Subsequently, the xerogels were immersed in an aqueous CPX solution (3 mg/mL, 5 mL) at 25 °C for 24 h, yielding dual-antibiotic PEGT_*x*_-GsCPX hydrogels. A representative image of the hydrogels after each step of preparation procedure is shown in Figure S1 in Supporting Information.

### 2.3 Characterization of Hydrogels

#### 2.3.1 Inverted Vial Test

Gelation time of PEGT_*x*_-Gs and PEGT_*x*_ hydrogels at 25 °C was qualitatively determined by the inverted vial test. The timer was started immediately after the addition of the DTT solution and vortex mixing. Gelation time was determined when no flow was observed after inverting the vial. The experiments were carried out in triplicate, so gelation times were presented as an average.

#### 2.3.2 Gel Fraction

PEGT_*x*_-Gs and PEGT_35_ hydrogels were lyophilized and weights of the resulting xerogels recorded as *W*_d_ (dried mass). The xerogels were then allowed to swell at 25 °C in Milli-Q water for 3 days with solvent being renewed every 24 h. The hydrogels were then lyophilized and the weights of the final xerogels (*W*_f_) were recorded again. All measurements were repeated in triplicate. The gel fraction was expressed as follows (mean ± SD, *n* = 3)

$$\mathrm{Gel}\,\;\mathrm{fraction}\;\left(\%\right)=\frac{W_{f}}{W_{d}}\times 100$$

#### 2.3.3 Attenuated Total Reflection (ATR) Infrared Spectroscopy

ATR spectra were recorded using a JASCO FT/IR-6600 spectrometer, with hydrogels being lyophilized before the experiment in order eliminate the aqueous media, obtaining the corresponding xerogel materials. For comparison purposes, the spectra were normalized by scaling the transmittance values to a range between 0 and 1 using the minimum and maximum transmittance values of each spectrum.

#### 2.3.4 Swelling Ratio

Hydrogels were frozen in liquid nitrogen and lyophilized for 24 h. The resulting xerogels were first weighed (dried mass, *W*_d_) and then immersed in PB at 37 °C. Swelling ratio was monitored by weighting, in doing so, the hydrogels were allowed to swell and their weight was recorded over time (*W_t_*), calculating their swelling ratio at each time as follows (mean ± SD, *n* = 3)

$$\mathrm{Swelling}\;\mathrm{Ratio}\;\left(\%\right)=\frac{W_{t}-W_{d}}{W_{d}}\times 100$$

#### 2.3.5 Rheological Characterization

PEGT_35_-Gs xerogels were swollen for 24 h at room temperature in either the CPX loading solution (yielding PEGT_35_-GsCPX swollen hydrogels) or phosphate buffer (PB, pH 7.4). After swelling, rheological measurements were performed with a HAAKE MARS 40 rheometer equipped with a parallel-plate measuring system. The swollen hydrogels were placed between the plates, and the storage (*G*′) and loss (*G*″) moduli were monitored at 37 °C, using a constant frequency of 0.1 Hz and a constant strain amplitude of 5%. For each sample, the reported *G*′ and *G*″ values correspond to the average of the eight consecutive measurements recorded during the time sweep. The final values are expressed as the mean ± SD of three independent samples.

#### 2.3.6 Morphology

The microstructure of the xerogels was visualized using Field Emission Scanning Electron Microscopy (FESEM, Carl Zeiss MERLIN). PEGT_*x*_-Gs and PEGT_*x*_-GsCPX hydrogels were prepared as described above and allowed to swell in PB for 24 h at 37 °C. Subsequently, hydrogels were frozen in liquid nitrogen and fractured to expose their cross sections. After lyophilization, the resulting xerogels were coated with Pt prior to observation. The pore size distributions were obtained through statistical analysis of the obtained images, for which an average of at least *n* = 50 pores was measured using ImageJ software.

#### 2.3.7 Hydrogel Degradation

Hydrogels after swelling-diffusion method in CPX aqueous solution (PEGT_*x*_-GsCPX) or after being under the conditions explained below to mimic the CPX-loading conditions (PEGT_*x*_-Gs, see Section 2.4.1) were weighted (*W*_0_) and immersed in 20 mL of buffer of the appropriate pH (7.4 or 5.0) at 37 °C. After 24 h of stabilization in the selected buffer, hydrogels were weighted again, using this weight as reference (*W*_ref_). Swelling/degradation was monitored by a weighting method, where the hydrogels were allowed to swell, and their weight was recorded at appropriate time points (*W*_*t*_). All measurements were repeated in triplicate. Results are represented as a function of the relative weight at each time (mean ± SD (*n* = 3))

$$\mathrm{Relative}\;\mathrm{weight}=\frac{W_{t}}{W_{\mathrm{ref}}}\times 100$$

### 2.4 Antibiotic Loading and Encapsulation Efficiency

#### 2.4.1 Gs Content Determination

PEGT_*x*_-Gs hydrogels were exposed to the same conditions used during CPX loading before determining the Gs content, to account for possible Gs loss associated with the CPX loading procedure. To do so, the hydrogels were first immersed in Milli-Q water (3 mL) for 4 h at 25 °C, with the aqueous medium renewed every hour. The hydrogels were then lyophilized overnight. Xerogels (in triplicate for each type of material) were soaked in diluted HCl aqueous solution (pH ∼ 4.5, 5 mL) at 25 °C for 24 h to mimic the CPX-loading conditions. To determine Gs entrapment, the residual Gs in the soaking solutions (Milli-Q water used for PB removal and HCl aqueous solution) was quantified using the OPA (*o*-phthalaldehyde) method.^41,42^ A derivatizing reagent was freshly prepared by dissolving *o*-phthalaldehyde (42 mg) in methanol (1 mL), followed by the addition of 2-mercaptoethanol (50 μL) and sodium borate buffer (9.3 mL, 0.04 M). After lyophilization of the Gs soaking solutions, equal volumes (2 mL each) of the derivatizing reagent, Gs solution in PB, and isopropanol (used to prevent precipitation) were mixed and incubated for 1 h at room temperature under orbital stirring. The absorbance of the resulting solution was then measured at 333 nm using a UV spectrophotometer. Based on the calibration curve (Figure S3a, Supporting Information), Gs content within the hydrogels was quantified. Drug loading (DL_GS_) and encapsulation efficiency (EE_GS_) were calculated as follows

$${\mathrm{DL}}_{\mathrm{GS}}\;\left(\%\right)=\frac{M_{0}-M_{s}}{M_{h}}\times 100$$

$${\mathrm{EE}}_{\mathrm{GS}}\;\left(\%\right)=\frac{M_{0}-M_{s}}{M_{0}}\times 100$$

where *M*_0_ represents the mass of Gs used for the preparation of the hydrogels, *M*_s_ represents the mass of Gs in soaking solutions after treatment of the hydrogels, and *M*_h_ represents the mass of the xerogels resulting from the lyophilization of PEGT_*x*_-Gs hydrogels. All measurements were performed in triplicate (mean ± SD (*n* = 3)).

#### 2.4.2 CPX Loading Determination

To determine CPX loading, PEGT_*x*_-GsCPX hydrogels were lyophilized, weighed (*M*_h_), and soaked in 6 M HCl solution (2 mL) to break the network and fully release the encapsulated CPX. The mixtures were incubated at room temperature under constant agitation for 24 h. Based on the calibration curve (Figure S3b, Supporting Information), the CPX loading amounts (*M*_*x*_) were quantified by absorbance at 271 nm using a UV–visible spectrophotometer. DL_CPX_ and EE_CPX_ and were calculated as follows

$${DL}_{CPX}\;\left(\%\right)=\frac{M_{x}}{M_{h}}\times 100$$

$${\mathrm{EE}}_{\mathrm{CPX}}\;\left(\%\right)=\frac{M_{x}}{M_{0}}\times 100$$

where *M*_0_ represents the initial mass of CPX in soaking solution before loading of hydrogels, *M*_*x*_ represents the mass of the CPX entrapped inside the hydrogel, and *M*_h_ represents the mass of the xerogel resulting from the lyophilization of CPX-loaded hydrogel. All measurements were performed in triplicate (mean ± SD (*n* = 3)).

### 2.5 Antibiotic Release Kinetics

#### 2.5.1 Gs Release Kinetics

*In vitro* release of Gs from PEGT_*x*_-Gs hydrogels was carried out at two different pH values (7.4 and 5.0). The hydrogels obtained after synthesis were subjected to post-treatment under the same conditions used for CPX loading (in triplicate for each type of material) and were subsequently individually immersed in 20 mL of buffer solution at 37 °C for up to 7 days. At predefined time points (1 h, 2 h, 4 h, 8 h, 1 day, 2 days, 3 days, 5 days, and 7 days, corresponding to *n* = 9 experiments), 1 mL of the release medium was withdrawn and replaced with an equal volume of fresh buffer. The collected aliquots were subsequently lyophilized, redissolved in 0.2 mL of Milli-Q water, and analyzed for Gs content using the OPA method (see above). Based on the calibration curve, the cumulative amount of Gs released over time (Cumulative Release, CR) was determined using the following equation

$$\mathrm{CR}\,\;\left(\%\right)=\frac{C_{n}V_{\mathrm{total}}+\sum_{i=1}^{n-1}C_{i}V_{i}}{M_{x}}\times 100$$

where *C*_n_ stands for drug concentration (mg/mL) in the release medium at the *n* experiment, *V*_total_ is the volume of the total release medium (20 mL), *C_i_* is drug concentration (mg/mL) in the *i*th withdrawn sample (for previous experiments to *n*), *V_i_* is the volume of the aliquot (1 mL) and *M*_*x*_ is the total mass of drug loaded into the hydrogel.

#### 2.5.2 CPX Release Kinetics

*In vitro* release of CPX from PEGT_*x*_-GsCPX hydrogels was performed at two different pH (7.4 and 5.0). PEGT_*x*_-GsCPX hydrogels (in triplicate for each type of material) were separately immersed in 20 mL of buffer solution at 37 °C for up to 7 days. At determined time intervals (1 h, 2 h, 3 h, 4 h, 6 h, 8 h, 1 day, 2 days, 3 days, 5 days, 7 days, corresponding to *n* = 11 experiments), a certain amount (1 mL) of the released medium was taken out, and an equal volume of fresh buffer was added in the original volume. Concentration of the aliquot was determined by measuring the absorbance at 271 nm using a UV–visible spectrophotometer. Based on the calibration curve, the cumulative release of CPX over time was determined using the previously shown equation.

#### 2.5.3 Mathematical Model Fitting

The Lindner-Lippold model was used to fit the release profiles of Gs and CPX, which is described by the equation^43^

$$\frac{M_{t}}{M_{\infty }}=kt^{n}+b$$

where *M*_*t*_ and *M*_∞_ represent the cumulative drug release at time *t* and at infinite time, respectively. The parameter *k* represents the kinetic constant of the process, *n* is the diffusional exponent that provides insight into the release mechanism, and *b* accounts for the burst release of the drug from the external part of the material. This model describes a diffusion-controlled drug release with an initial burst release effect. Other mathematical models were applied so that the best-fitting equation was determined by evaluating the coefficient of determination (*R* ^2^) obtained for each case (Figures S6 and S7, Supporting Information).

### 2.6 *In Vitro* Biological Evaluation

#### 2.6.1 Minimum Inhibitory Concentration (MIC) and Minimum Bactericidal Concentration (MBC) Determination of Free Drugs

The antibacterial activity of free antibiotics was individually evaluated against MRSA and *P. aeruginosa*. Both bacterial strains were cultured overnight in TSB at 37 °C with continuous shaking at 150 rpm until reaching the stationary growth phase. Stock solutions of the antibiotics were prepared in sterile water, diluted in TSB to the desired test concentrations, and then mixed with bacterial inocula to achieve a final bacterial concentration of 10^5^ Colony Forming Units per mL (CFU/mL). After 24 h of incubation at 37 °C with shaking at 150 rpm, bacterial viability was assessed using the standard serial dilution method. Briefly, samples were diluted in DPBS and spot-plated onto TSA plates. Colonies present in plates were counted after 24 h of incubation at 37 °C. Positive control samples (untreated bacteria) were included in each assay. All experiments were conducted in triplicate, with three replicas each (*n* = 9).

#### 2.6.2 Fractional Inhibitory Concentration Index (FICI) Determination

FICI was determined by the checkerboard assay in order to evaluate *in vitro* interactions of both antibiotics. For each strain of bacteria (MRSA and *P. aeruginosa*), stock solutions containing four times the individual MIC of both antibiotics were prepared. In 96-well plates, each antibiotic was diluted 2-fold vertically and horizontally, respectively. Then, 20 μL of bacteria suspensions were added in each well achieving a final concentration of 10^5^ CFU/mL, using a bacteria-free mirror plate as negative control. The plates were then incubated at 37 °C for 24 h. Turbidity was observed by naked eye inspection and quantified by measuring the absorbance at 600 nm using a Varioskan LUX microplate reader (Thermo Scientific, Waltham, USA). The assay was performed in triplicate (*n* = 3). FICI index was calculated using the following equations

$$\mathrm{FICI}={\mathrm{FICI}}_{\mathrm{CPX}}+{\mathrm{FICI}}_{\mathrm{Gs}}$$

Where

$${\mathrm{FICI}}_{\mathrm{CPX}}=\frac{{\mathrm{MIC}}_{\mathrm{CPX}}\;\mathrm{in}\;\mathrm{presence}\;\mathrm{of}\;\mathrm{Gs}}{{\mathrm{MIC}}_{\mathrm{CPX}}}$$

$${\mathrm{FICI}}_{\mathrm{Gs}}=\frac{{\mathrm{MIC}}_{\mathrm{Gs}}\;\mathrm{in}\;\mathrm{presence}\;\mathrm{of}\;\mathrm{CPX}}{{\mathrm{MIC}}_{\mathrm{Gs}}}$$

Antibiotic interactions were identified as synergy (FICI < 0.5), addition (0.5 ≤ FICI ≤ 1), indifference (1 < FICI ≤ 4) or antagonism (FICI > 4).^12^

#### 2.6.3 MIC and MBC Determination of PEGT_35_-GsCPX

MIC and MBC values for PEGT_35_-GsCPX hydrogels’ supernatants were evaluated. Briefly, lyophilized hydrogels or xerogels were cut into small pieces (0.1–4 mg) and placed into 10 mL sterile Falcon tubes after UV sterilization for 30 min. Each tube was then filled with 2 mL of TSB and incubated at 37 °C for 24 h to allow drug release. Subsequently, 0.5 mL of the release medium, either undiluted (for MRSA experiments) or diluted 1:100 (for *P. aeruginosa* experiments), was inoculated with the selected bacterial strain at a final concentration of 10^5^ CFU/mL. After 24 h of incubation at 37 °C with shaking at 150 rpm, bacterial viability was determined using the standard serial dilution method. Samples were diluted in DPBS and spot-plated onto TSA plates. Colonies were counted after 24 h of incubation at 37 °C. Positive control samples (untreated bacteria and bacteria treated with supernatants from unloaded PEGT_35_ hydrogels at MIC and MBC concentrations) were included. All experiments were conducted in triplicate, with three replicas each (*n* = 9).

#### 2.6.4 Cytotoxicity Evaluation on Eukaryotic Cells

To determine the cytotoxic effect of PEGT_35_-GsCPX hydrogels’ supernatants, human dermal fibroblasts (NHDF-Ad) and J774 murine macrophages were used as model cell lines. All cell lines were grown in high-glucose Dulbecco’s modified Eagle’s medium (DMEM w/stable glutamine) supplemented with 10% (v/v) FBS and 1% (v/v) antibiotic-antimycotic solution at 37 °C and 5% CO_2_. The Blue Cell Viability Assay was used to measure the cell metabolism associated with cell viability. PEGT_35_-GsCPX xerogels were cut into small pieces (0.1–4 mg) and were allowed to release in cell culture medium for 24 h at 37 °C at different concentrations, mimicking those used for the MIC and MBC determination assays (see above). Fibroblasts and J774 macrophages were seeded in the inner wells of 96-well plates at a density of 2 × 10^4^ cells/cm^2^. After 24 h at 37 °C, the medium was aspirated, the wells were washed with DPBS, and 100 μL of the xerogel supernatants were added. Cells were then incubated again for 24 h at 37 °C, and then the viability assays were performed. The medium was aspirated, the wells were washed with DPBS, and 100 μL of Blue Cell Viability reagent (10% v/v in DMEM) was added following the manufacturer’s instructions, followed by incubation for 3 h at 37 °C, 5% CO_2_. Fluorescence was then measured (528/590 nm excitation/emission). Cytotoxicity was evaluated by comparing the fluorescence values of treated cells with those of untreated control cells (set as 100% viability). Cytotoxicity evaluation of supernatants from unloaded PEGT_35_ hydrogels at the maximum concentration tested were included as control. All experiments were conducted in triplicate, with three replicas each (*n* = 9).

## 3 Results and Discussion

### 3.1 Synthesis and Characterization of Dual-Antibiotic Hydrogels

Dual-antibiotic hydrogels were prepared through two-step process: first, Gs was covalently incorporated during network formation; second, CPX was physically loaded via a swelling-diffusion method (Figure 1a,b, respectively). Initially, Gs, which contains five amino groups capable of β-aminoacrylate formation, was directly used to form hydrogels by mixing PEGalk (molar mass 10 000 g/mol) and Gs in phosphate buffer (PB, pH 7.4), using a 1:1 molar ratio of alkynoate to amine groups and varying the polymer concentration (denoted as %PEG-Gs hydrogels in Table S3, Supporting Information). Only hydrogels reaching 20 and 30% of polymer concentration were formed, for both of which gelation occurred within 12–18 h (overnight) at 25 °C. It is worth noting that commercially available Gs comprises five closely related congeners, so that each one bears three or four primary amino groups and two or one secondary amino groups.^44^ Therefore, these results are consistent with our previously reported data on model compounds, where amino-yne hydrogels formed predominantly through primary amines generally require high polymer concentrations to induce gelation, presumably due to their lower nucleophilicity compared to secondary amines.^29^ However, efforts to incorporate CPX within the network of these materials were unsuccessful. When CPX was introduced *in situ* during hydrogel formation, its low solubility at physiological pH led to precipitation, thereby preventing network formation. Moreover, hydrogels were unable to swell in the presence of CPX without undergoing significant degradation, which can be attributed to the acidic pH of the CPX aqueous solution (pH ∼ 4.5), as it may induce β-aminoacrylate cleavage and consequently weaken the hydrogels. Therefore, to enhance the mechanical integrity and modulate the hydrogel degradability required for CPX loading, DTT was incorporated into the formulation, as β-thioacrylate linkages are expected to confer greater stability to the hydrogels and a slower response to acidic environments.

Then, as schematized in Figure 2a, Gs-conjugated hydrogels were synthesized by combining PEGalk, which acted as the scaffold for network formation, through two types of Michael additions: spontaneous amino-yne and thiol-yne reactions. Covalent cross-links were formed when the amines of Gs and thiols of DTT react with the propiolate groups of PEGalk, yielding β-aminoacrylate and β-thioacrylate linkages, respectively. A series of three hydrogels was prepared by fixing PEGalk at a polymer concentration of 20 wt %, maintaining a 1:1 molar ratio of alkynoate to amino groups of the Gs and varying DTT concentration (15, 25, and 35% mmol of thiol groups with respect to mmol of alkynoate groups), in order to investigate how dithiol cross-linker (DTT) content influences hydrogel properties and performance as drug delivery system. Accordingly, the resulting hydrogels were denoted as PEGT_*x*_-Gs, where *x* represents the mol % of thiol groups (T_*x*_) relative to the alkynoate groups used during network formation. For hydrogel preparation, PEGalk was weighed and dissolved in PB under orbital stirring for 4 h at room temperature. Gs and DTT were separately dissolved in the same buffer, at the appropriate concentration to achieve the desired alkynoate:amine and alkynoate:thiol ratios, maintaining a final hydrogel mass of 100 mg (PEGalk + PB) in all cases. Then, the Gs solution was added and allowed to react for 1 h at 25 °C prior to DTT addition. The mixtures were subsequently maintained at 25 °C for 18 h to complete hydrogel formation, yielding self-standing hydrogels (Figure 2b; see the Section 2.2 and Table S1 in Supporting Information for details on hydrogel composition). Sequential addition of nucleophiles was selected to favor amine addition over thiol addition, as amines are weaker nucleophiles than thiols, thereby improving Gs conjugation through greater availability of propiolate groups. A reaction time of 1 h was selected for Gs, as longer reaction times resulted in highly viscous solutions that hindered efficient mixing with DTT. Inverted vial tests were performed to qualitatively determine the gelation time of hydrogels with varying mol % of thiol groups. Time counting was started immediately after the addition of the DTT solution, and the gelation times are summarized in Figure 2c. As expected, increase in the amount of DTT induced shorter gelation times, decreasing from approximately 12–18 h (overnight) in the case of PEGT_15_-Gs, to 210 and 26 s for PEGT_25_-Gs and PEGT_35_-Gs, respectively. To further evaluate the efficiency of the cross-linking process, gel fraction tests (see Section 2.3 for details) were conducted to quantify the portion of material forming an insoluble network. The as prepared materials were lyophilized and subsequently allowed to swell in Milli Q water for 3 days, with frequent water exchanges to remove unreacted compounds. As shown in Figure 2d, all PEGT_*x*_-Gs samples exhibited gel fraction values over 70%, which is in line with those reported in the literature for thiol-yne-based hydrogels.^32^ Moreover, they demonstrated dependence on the dithiol content, with hydrogels containing higher DTT cross-linker amounts exhibiting increased gel fraction values, reaching up to 83% for PEGT_35_-Gs.

**Figure 2:**
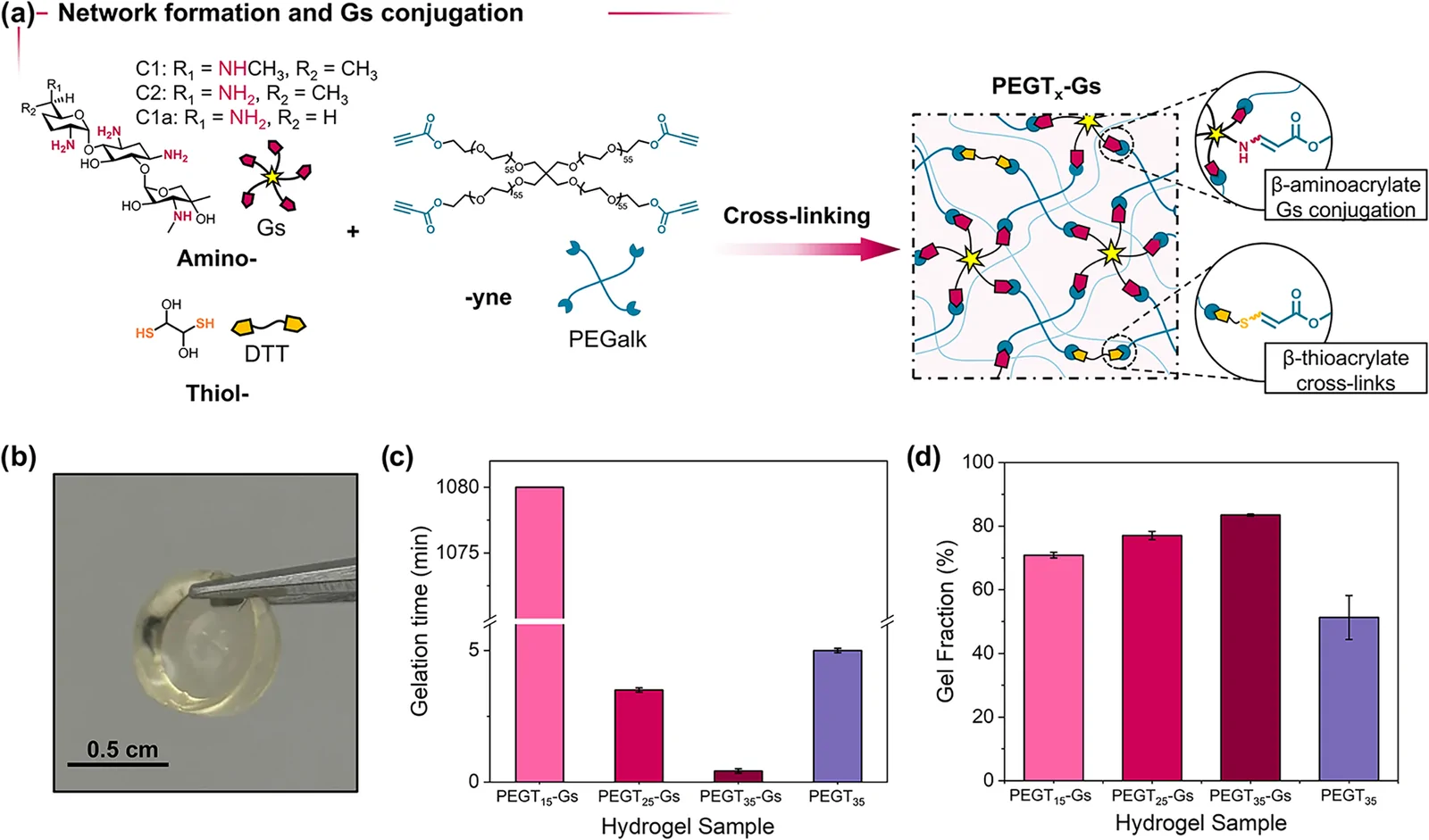
(a) Synthesis of PEGT_*x*_-Gs hydrogels through spontaneous amino-yne and thiol–yne cross-linking of PEGalk with Gs and DTT, respectively. The molecular structure of Gs is represented showing three of its most abundant gentamicin C congeners.^44^ (b) Photograph of self-standing PEGT_35_-Gs hydrogel. (c) Gelation time and (d) gel fraction of PEGT_*x*_-Gs and PEGT_35_ hydrogels.

With the aim of comparison, hydrogels were also prepared using PEGalk and DTT (15, 25, or 35 mol % of thiol groups) without the addition of Gs. Gelation was only observed at the highest cross-linker content (PEGT_35_, see preparation details in Table S2, Supporting Information), with gelation time of approximately 300 s. Besides, PEGT_35_ exhibited a low gel fraction (approximately 50%, Figure 2d), which is consistent with the fact that only 35 mol % of thiol groups were added relative to the total alkynoate groups, resulting in incomplete network formation and a significant fraction of unreacted polymer chains. These results demonstrate the occurrence of the amino-yne reaction and highlight its importance in the hydrogel network formation.

In the second step of material preparation, CPX was physically loaded into the PEGT_*x*_-Gs hydrogels using a swelling-diffusion method (Figure 3a). Briefly, the as-prepared hydrogels were immersed in Milli-Q water for 4 h at 25 °C to remove the PB retained within the network, thereby preventing CPX precipitation during the swelling and loading, due to its low solubility at physiological pH. The subsequently lyophilized xerogels (dimensions reported as length × width × height) measured approximately 1.4 cm × 1.4 cm × 2 mm, 1.1 cm × 1.1 cm × 4 mm and 0.9 cm × 0.9 cm × 4 mm for PEGT_15_-Gs, PEGT_25_Gs and PEGT_35_Gs, respectively (see Figure S1 in the Supporting Information). The xerogels were then immersed in an aqueous CPX solution (3 mg/mL, pH ∼ 4.5) and allowed to swell for 24 h at a controlled temperature of 25 °C. The resulting dual-antibiotic hydrogels were used without further treatment and will hereafter be referred to as PEGT_*x*_-GsCPX.

**Figure 3:**
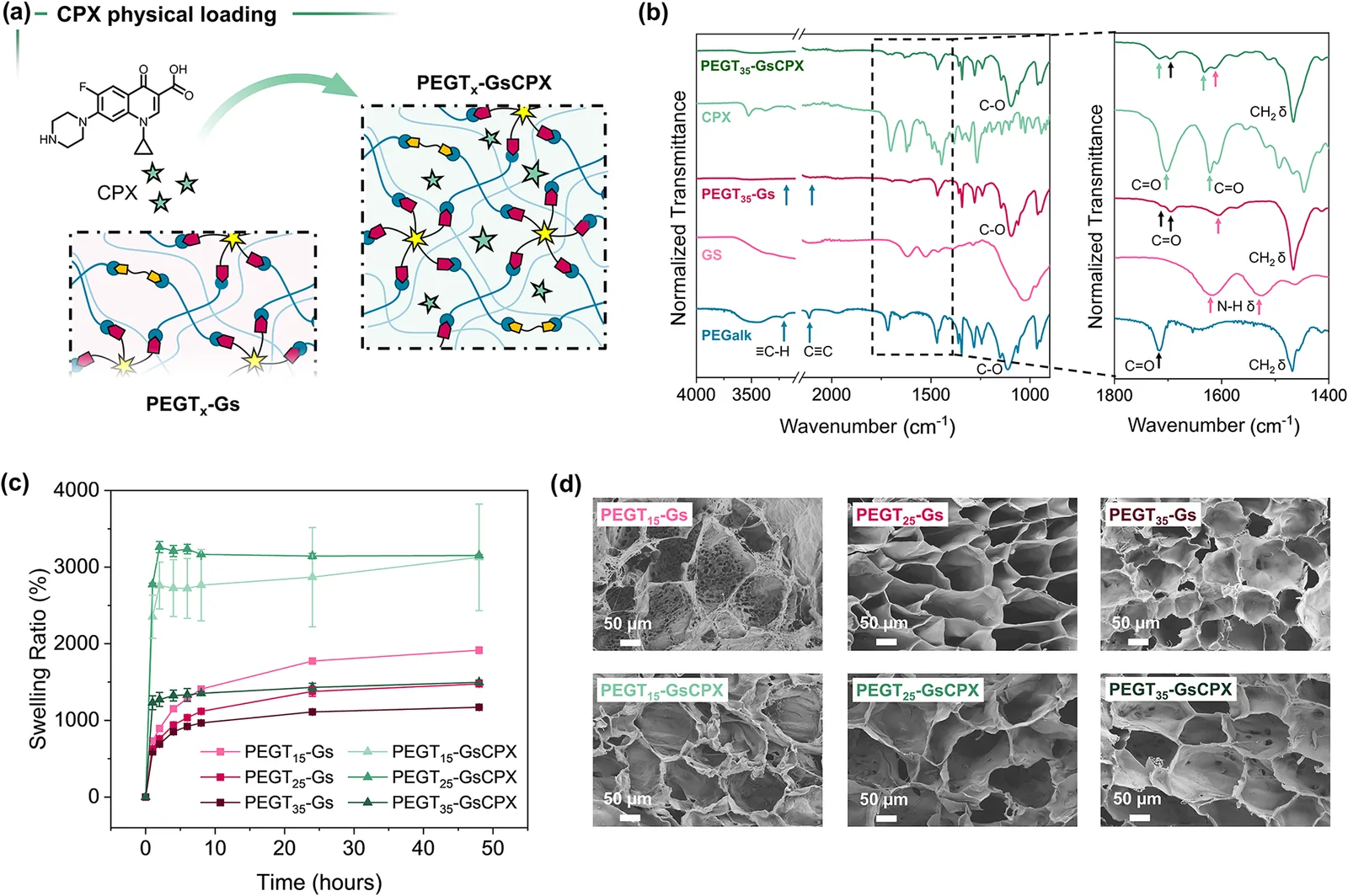
(a) CPX loading through swelling-diffusion method. (b) From bottom to top: ATR spectra of PEGalk, Gs, PEGT_35_-Gs xerogel, CPX and PEGT_35_-GsCPX xerogel (left) and magnification of the spectra from 1800 to 1400 cm^–1^ (right). (c) Swelling ratio of PEGT_*x*_-Gs (pink) and PEGT_*x*_-GsCPX (green) hydrogels. (d) FESEM images of the cross-sectional morphology of PEGT_*x*_-Gs and PEGT_*x*_-GsCPX xerogels after 24 h immersed in PB at 37 °C.

Chemical structure of the materials was studied using ATR infrared spectroscopy. Taken as an example, PEGT_35_-Gs, vibrations associated with the polymeric network were observed as two weak bands at 1714 and 1695 cm^–1^ (C=O stretching, Figure 3b, black arrows), and more intense bands at 1460 cm^–1^ (CH_2_ δ) and at 1093 cm^–1^ (C–O stretching). Characteristic bands of the propiolate groups in PEGalk (blue arrows in Figure 3b, ≡C–H and C≡C stretching vibrations at 3222 and 2112 cm^–1^, respectively) were not detected in PEGT_35_-Gs hydrogel sample, indicating efficient Michael addition and network cross-linking. In addition, the spectrum of hydrogels prepared exclusively from PEGalk and Gs (20PEG-Gs) showed a marked reduction in the intensity of the characteristic alkyne bands compared with that of PEGalk, providing further evidence of Gs conjugation (see the expanded ≡C–H stretching region in absorbance mode in Figure S2, Supporting Information). The spectrum of free Gs showed bands at 1620 and 1529 cm^–1^ assigned to the N–H bending vibration of primary amines (Figure 3b, pink arrows),^45^ whereas in PEGT_35_-Gs only the most intense band was observed at 1605 cm^–1^, which shifted to lower wavenumber. In the spectrum of PEGT_35_-GsCPX, two bands at 1717 and 1632 cm^–1^ confirmed the presence of CPX within the network. These bands appeared slightly shifted compared to those of free CPX, observed at 1701 and 1620 cm^–1^ (Figure 3b, green arrows), corresponding to the carbonyl groups of the carboxylic acid and the pyridone moieties, respectively.^46^

Swelling capacity is one of the most significant inherent characteristics of hydrogels for wound healing, since it affects both the absorption of exudates at the wound surface and drug release efficiency.^47^ Accordingly, swelling ratio of the materials was determined (see details in Section 2.3.4) under physiological conditions (PB at pH = 7.4, 37 °C), before and after CPX loading. Upon immersion into the PB buffer, the non-CPX loaded PEGT_*x*_-Gs hydrogels exhibited a progressive swelling behavior, reaching equilibrium after 24 h (Figure 3c, pink lines). The equilibrium swelling ratio values decreased with increasing dithiol cross-linker content, which is in accordance with previous literature,^48^ from 1800% for PEGT_15_-Gs to 1100% for PEGT_35_-Gs. After CPX loading, the swelling of the hydrogels was notably faster, reaching the plateau within 1–2 h of aqueous immersion (Figure 3c, green lines). Moreover, the equilibrium swelling ratio values of hydrogels with the same cross-linker percentage increased markedly after CPX loading. This effect was more pronounced at lower cross-linker content, with PEGT_15/25_-GsCPX showing a 2–2.5-fold increase in its equilibrium swelling ratio after loading, compared to the 1.2-fold increase observed for PEGT_35_-GsCPX. Besides, PEGT_15_-GsCPX hydrogels showed rounded edges and partial breakage upon handling during swelling, together with a high variability in the experimental results. This behavior could be attributed to partial acid-promoted cleavage of β-aminoacrylate bonds during CPX loading, due to the acidic pH of the CPX solution, leading to partial degradation of the hydrogel. To further evaluate whether the acidic CPX loading procedure affected the hydrogel integrity, additional rheological measurements were performed on PEGT_35_-GsCPX hydrogels after swelling in either the CPX solution (pH ∼ 4.5) or PB (pH 7.4) for 24 h at 25 °C (see Section 2.3.5 for details). As shown in Table S4, the CPX-loaded hydrogel exhibited *G*′ and *G*″ values of 13.0 ± 2.1 and 0.8 ± 0.2 kPa, respectively, while the PB-swollen hydrogel showed *G*′ and *G*″ values of 15.3 ± 0.7 and 1.7 ± 0.4 kPa, respectively. Although the moduli decreased after CPX loading, the differences were small, indicating that the loading procedure does not substantially compromise the mechanical integrity of the hydrogel.

The cross-sectional morphologies of the materials were observed using FESEM. To assess whether CPX loading also affected the internal morphological structure of the materials, hydrogels before (PEGT_*x*_-Gs) and after CPX loading (PEGT_*x*_-GsCPX) were swollen in PB for 24 h, then frozen in liquid nitrogen, fractured to expose the cross-section, and subsequently lyophilized. As shown in Figure 3d, all the resulting xerogels exhibited characteristic interconnected porous structures. Their pore size slightly decreased with increasing contents of β-thioacrylate cross-links, ranging approximately from 105 ± 22 μm for PEGT_15_-Gs to 94 ± 33 μm for PEGT_35_-Gs, attributable to the increased cross-linking density. Only minor changes were observed after loading CPX, with an increase in pore size and heterogeneity (see Table S5 in Supporting Information).

### 3.2 Antibiotic Quantification and Release Studies

Quantification of Gs and CPX within the hydrogel network was performed by UV–vis spectroscopy (see Section 2.4), and the results are summarized in Table 1. To determine Gs content, samples were derivatized with the OPA method in the presence of a thiol, following previously reported procedures,^41,42^ and the resulting derivatives were quantified by UV detection at 333 nm (see Section 2.4 for details). Quantification of Gs in the presence of CPX proved challenging due to overlapping UV spectra and the higher absorption intensity of CPX, as well as the poor solubility of CPX in neutral or basic media, a condition required for the use of OPA and other derivatization methods. Consequently, Gs quantification was carried out using CPX-free PEGT_*x*_-Gs hydrogels, which were processed under the same methodology and conditions as those used for CPX-loaded samples (*i.e.*, PB removal and immersion in acidic solution, pH ∼ 4.5, for 24 h; see Section 2.2). This procedure, in addition to reproducing the CPX loading conditions, allowed the elimination of any Gs that might remain physically entrapped within the hydrogel network after gelation, as well as accounting for any Gs release associated with partial cleavage of β-aminoacrylate bonds to a limited extent during CPX loading, as inferred from swelling experiments. In this way, the drug loading (DL_Gs_) and encapsulation efficiency (EE_Gs_) values reflect the actual fraction of Gs covalently bound to the network, which should be higher in the absence of this post gelation treatment. Obtained values were DL_Gs_ = 3.8–3.2 wt % and EE_Gs_ = 55.2–62.9 wt %, with only minor differences observed between the lowest and highest cross-linked hydrogels.

**Table 1:** Drug Loading (DL, wt %) and Encapsulation Efficiency (EE, wt %) of Gs and CPX in Treated PEGT_*x*_-Gs and PEGT_*x*_-GsCPX Hydrogels, Measuring Approximately 1.8 cm × 1.8 cm × 2 mm, 1.3 cm × 1.3 cm × 5 mm and 1.1 cm × 1.1 cm × 5 mm for PEGT_15_-GsCPX, PEGT_25_GsCPX and PEGT_35_GsCPX, Respectively (Dimensions Reported as Length × Width × Height, See Figure S1 in the Supporting Information)

Sample	Antibiotic	DL (%)	EE (%)
PEGT_15_-GsCPX	Gs	3.8 ± 0.3	55.2 ± 4.2
	CPX	6.8 ± 0.1	6.8 ± 0.2
PEGT_25_-GsCPX	Gs	3.3 ± 0.5	59.6 ± 6.2
	CPX	5.3 ± 0.2	6.3 ± 0.2
PEGT_35_-GsCPX	Gs	3.2 ± 0.4	62.9 ± 6.0
	CPX	4.6 ± 0.1	5.8 ± 0.2

CPX content was determined directly by measuring the absorbance at 271 nm, corresponding to its absorption maximum. CPX drug loading (DL_CPX_) values ranged from 6.8 wt % for PEGT_15_-GsCPX to 4.6 wt % for PEGT_35_-GsCPX, decreasing with increasing cross-linker content, due to a reduction in the swelling capacity of the hydrogel network, as previously discussed. These values were similar to those reported in literature for CPX physically loaded in hydrogels.^49,50^ Encapsulation efficiency (EE_CPX_) values followed the same trend, decreasing from 6.8 to 5.8 wt %. The low EE_CPX_ values may be attributed either to a reduced CPX solubility within the network, or to an excess amount of CPX used during the loading process.

The dual and pH-dependent release of Gs and CPX was evaluated as a function of cross-linking degree and pH at 37 °C. Phosphate buffer (PB, pH = 7.4, 0.1 M) was used to mimic physiological conditions of an open wound, whereas sodium acetate buffer (pH = 5.0, 0.1 M) was employed to study the effect of acid-induced β-aminoacrylate cleavage on the release profile, simulating the environment associated with bacterial growth during early stages of wound healing.^14,51^

As shown in Figure 4, the cumulative release profiles of CPX (green) and Gs (pink) at pH = 7.4 and pH = 5.0 over 7 days displayed two different behaviors depending on their incorporation mode. CPX, which was physically entrapped within the hydrogel, was rapidly released by diffusion, whereas Gs covalently conjugated to the hydrogel matrix showed a slower release as expected. At physiological pH, the dithiol cross-linker content (15, 25, or 35%) primarily governed the early stage of CPX release (Figure 4a–c, green solid lines). The least cross-linked sample, PEGT_15_-GsCPX showed the fastest release, reaching 89% within the first 8 h, while the most cross-linked system, PEGT_35_-GsCPX, reached 71% over the same period. PEGT_25_-GsCPX exhibited intermediate cumulative release values, although the differences between PEGT_25_-GsCPX and PEGT_35_-GsCPX were relatively small, suggesting a reduced effect of cross-linker content at higher values. In all cases, CPX release rate markedly decreased after this initial release stage (6–8 h), transitioning into a slower diffusion regime that approached a plateau, with overall release largely completed within the first 24 h (92–95%).

**Figure 4:**
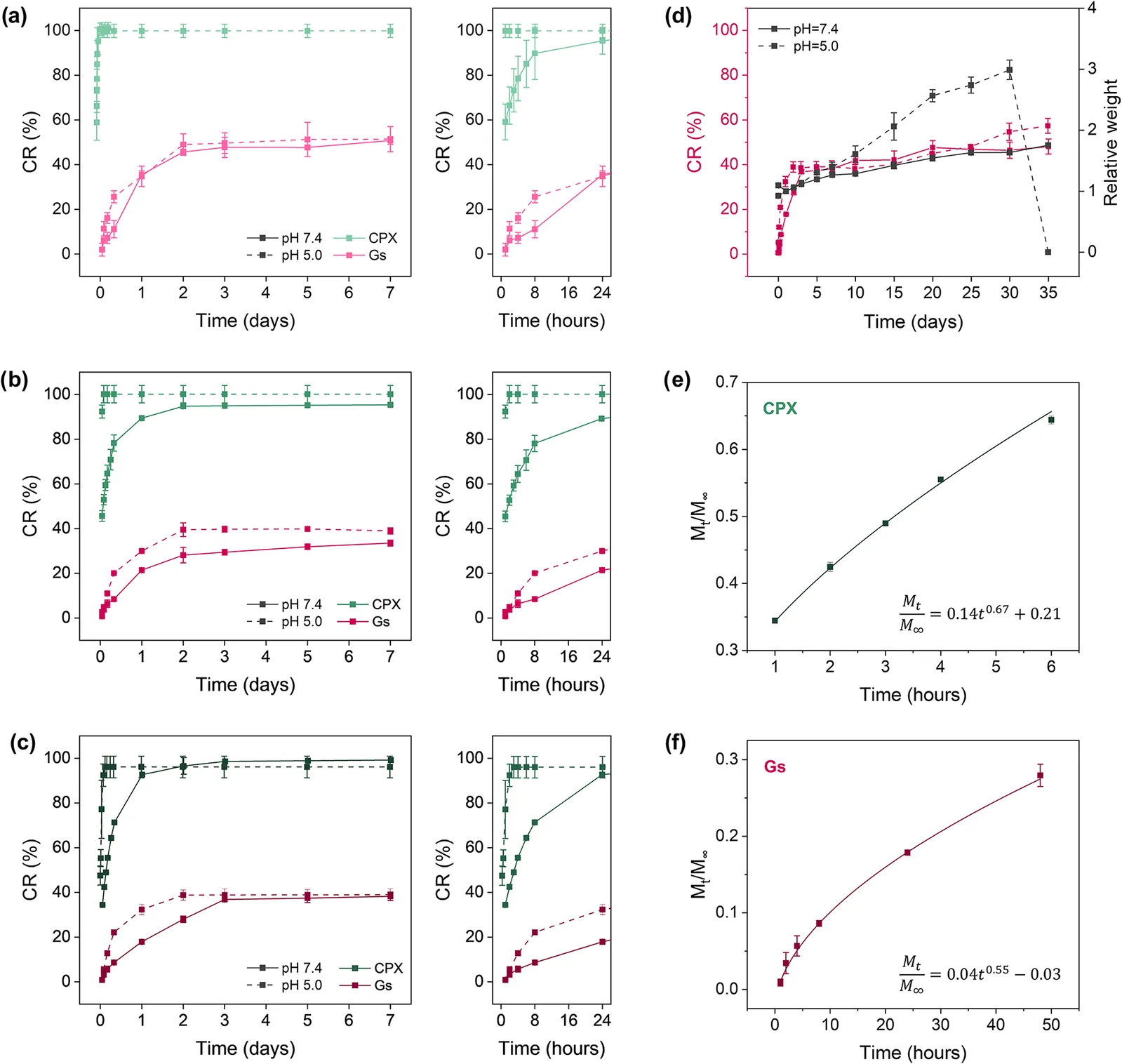
Cumulative release (CR, %) profiles over 7 days (left) and magnification of the first 24 h (right) of CPX (green) and Gs (pink) at pH = 7.4 (solid lines) and pH = 5.0 (dashed lines) from (a) PEGT_15_-GsCPX or PEGT_15_Gs, used as reference to study Gs release, (b) PEGT_25_-GsCPX or PEGT_25_Gs and (c) PEGT_35_-GsCPX or PEGT_35_Gs hydrogels. (d) CR of Gs (pink lines) and relative weight of PEGT_35_-Gs hydrogels (black lines) over 35 days at pH = 7.4 (solid lines) and pH = 5.0 (dashed lines); the abrupt decrease observed on day 35 is attributed to hydrogel solubilization between days 30 and 35. (e) Data fitting of drug release by Lindner-Lippold model equation for CPX from PEGT_35_-GsCPX hydrogels and (f) for Gs from PEGT_35_-Gs hydrogels.

For Gs (Figure 4a–c, pink solid lines), a distinct release behavior was also observed, with an even stronger dependence on the cross-linker content. Gs release also exhibited an initial faster release, followed by a marked decrease in release rate and an approach to a plateau. However, the overall extent of release was significantly lower than that of CPX, with a maximum cumulative release of 38–51% after 7 days, depending on the thiol cross-linker content. For the least cross-linked hydrogel, PEGT_15_-Gs, approximately 35% of Gs was released within 24 h, reaching 51% at 7 days. In the sample cross-linked the most, PEGT_35_-Gs, release was more sustained, with only 18% released at 24 h, and 36% reached at 72 h, with a slight increase thereafter. Again, PEGT_25_-Gs showed an intermediate profile. Overall, increasing β-thioacrylate linkages leads not only to reduced release rates but also to a lower cumulative Gs release at later stages of the release profile, suggesting that a denser network limits both CPX diffusion-controlled release and the water penetration required for the hydrolytic cleavage of β-aminoacrylate linkages responsible for Gs release.

Under acidic conditions, CPX release exhibited a pronounced burst effect, with complete drug release achieved within 1, 2, or 3 h for PEGT_15_-GsCPX, PEGT_25_-GsCPX and PEGT_35_-GsCPX, respectively (Figure 4a–c, green dashed lines). These results are consistent with previous studies reporting higher CPX solubility at pH = 5.0 compared to that at pH 7.4,^52^ which facilitates diffusion into the surrounding medium and, consequently, faster release. For Gs, the release profile was also faster than that at physiological pH, with the most notable differences observed in the hydrogel cross-linked the most (Figure 3c, pink dashed lines). Specifically, PEGT_35_-Gs released almost twice as much Gs within the first 24 h compared to the amount released at pH = 7.4 (32% versus 18%), followed by a moderate increase to 39% after 48 h. In this case, since Gs exhibits high solubility under both physiological and acidic conditions, the faster release kinetics can be attributed to the acidic pH-induced β-aminoacrylate cleavage between Gs and the polymer matrix. Therefore, these results highlight that the release rate of the antibiotics can be adjusted both by controlling the cross-linking density through the incorporation of β-thioacrylate linkages, and by variations in the pH of the surrounding microenvironment.

Long-term Gs release studies were conducted over 35 days under both physiological (pH = 7.4) and acidic (pH = 5.0) conditions for PEGT_35_-Gs hydrogels, while simultaneously monitoring the relative hydrogel weight to evaluate swelling and degradation of the system (Figure 4d, see Section 2.4 for details). Complete Gs release was not achieved under either condition, reaching 57% at pH = 5.0 and 48% at pH = 7.4 after 35 days. Despite this, the hydrogel incubated at pH = 5.0 underwent almost complete solubilization by the 35th day, whereas the hydrogel maintained at pH = 7.4 remained stable throughout this period, with no evidence of degradation even after 60 days. Therefore, incomplete recovery of Gs may be attributed to the complex degradation pathways occurring over extended time scales, where hydrogel degradation involves not only β-aminoacrylate cleavage but also ester hydrolysis,^29^ leading to heterogeneous degradation products. Consequently, not all amino groups of Gs may be available for derivatization and detection by the OPA method, which likely contributes to the incomplete quantification of the antibiotic even after apparent macroscopical hydrogel degradation. In order to verify that PEGT_35_-Gs hydrogels effectively mimic the macroscopic behavior of the CPX-loaded systems, swelling and degradation experiments were repeated using PEGT_35_-GsCPX, which showed very similar swelling and degradation trends (Figure S4, Supporting Information). Moreover, PEGT_15_-GsCPX and PEGT_25_-GsCPX hydrogels were also subjected to degradation studies, with the sample containing the lowest cross-linker content showing premature solubilization compared to the other formulations (Figure S5, Supporting Information).

To better understand the release kinetics and transport mechanisms involved in drug release, different mathematical models were used to fit the experimental data (Figures S6 and S7, Supporting Information). It is important to note that these models are generally considered valid only as short-time approximations, typically up to 60% of the cumulative drug release.^53^ Since more than 60% of CPX release is reached within only 1 and 3 h for PEGT_15_-GsCPX and PEGT_25_-GsCPX, respectively, the number of data points available seemed insufficient. Therefore, only the experimental data from PEGT_35_-GsCPX were used for mathematical models. Moreover, for Gs, whose cumulative release after 1 week was 38%, the mathematical models were applied directly to the experimental data up to 60% of the maximum drug released, as proposed by other authors.^54^ Here, the Lindner-Lippold model (see Section 2.5 and Figure 4e,f) provided the highest coefficients of determination for both CPX and Gs release (*R* ^2^ = 0.997 and 0.999, respectively). The higher kinetic constant observed for CPX compared to Gs (*k* = 0.14 and 0.04, respectively) indicated a faster overall release rate of the physically entrapped drug. Regarding the diffusion exponent *n*, deviations from ideal Fickian diffusion (*n* = 0.45 for cylindrical geometry) were observed, with values falling within the anomalous transport range (0.45 < *n* < 0.89; *n* = 0.67 for CPX and *n* = 0.55 for Gs). This indicated that release was governed not only by diffusion but also by contributions from the polymeric network, such as swelling, chain relaxation, or erosion.^55,56^ The *b* parameter further differentiated the two systems: CPX exhibited a positive value (*b* = 0.21), consistent with a noticeable initial burst release, whereas Gs showed a slightly negative value (*b* = −0.03), indicating that the contribution of burst release from the surface was negligible.^57^ The difference in release kinetics between CPX and Gs therefore supports their distinct modes of encapsulation. While CPX presents a faster release with an initial burst phase, the covalent conjugation of Gs within the hydrogel matrix enables slower release and, consequently, a potential prolonged antimicrobial effect.

### 3.3 *In Vitro* Biological Evaluation

The bactericidal activity of the free antibiotics was first tested, using MRSA and *P. aeruginosa* planktonic cultures as Gram-positive and Gram-negative models, respectively. MIC and MBC obtained values by the serial dilution method (see Section 2.6) are shown in Table 2 and Figure S8 (Supporting Information). For Gs, a broad-spectrum antibiotic active against both Gram-positive and Gram-negative bacteria, the concentrations required to inhibit bacterial growth or achieve bactericidal effect were low and similar for MRSA (MIC = 1 μg/mL, MBC = 2 μg/mL) and *P. aeruginosa* (MIC = 1 μg/mL, MBC = 4 μg/mL), in agreement with previously published data.^58,59^ Results obtained for CPX against MRSA (MIC = 16 μg/mL, MBC > 356 μg/mL) were high but consistent with those previously reported in the literature.^60^ CPX is known to be particularly potent against Gram-negative organisms;^34^ accordingly, the values obtained against *P. aeruginosa* were markedly lower (MIC = 0.1 μg/mL, MBC = 1 μg/mL). Then, *in vitro* antibiotic interactions were evaluated by calculating the FICI, which enables classification of the interaction between the two agents (see Section 2.6). For both bacterial strains, obtained FICI values of 1 indicated an additive effect; that is, the combination of each antibiotic at half of its MIC produced the same inhibitory effect as the MIC of each antibiotic used alone (Table 2). Importantly, it suggests that the combination of Gs and CPX, when coloaded into the system, can inhibit the growth of both MRSA and *P. aeruginosa* at lower concentrations than when each antibiotic is used independently.

**Table 2:** Antibacterial Activity as MIC and MBC of Individual Free Antibiotics Gs and CPX, Free Antibiotics When Added in Combination (Gs-CPX) and in Loaded Xerogels (PEGT_35_-GsCPX)

		MRSA		*P. aeruginosa*	
Sample		MIC (μg/mL)	MBC (μg/mL)	MIC (μg/mL)	MBC (μg/mL)
Free antibiotics	Gs	1	2	1	4
	CPX	16	>356	0.1	1
	Gs-CPX	Gs 0.5 CPX 8	-	Gs 0.5 CPX 0.05	-
PEGT_35_-GsCPX	Xerogel^a^	135	200	2.5–5	20
	Released antibiotics^b^	Gs 0.78 CPX 5.7	Gs 1.2 CPX 8.5	Gs 0.014–0.03 CPX 0.11–0.21	Gs 0.11 CPX 0.85

a PEGT_35_-GsCPX xerogel-to-medium mass:volume ratio (μg:mL).

b Released concentration of each antibiotic after 24 h, according to cumulative release studies shown in Figure 4c, is presented as reference.

After evaluating the activity of the free antibiotics, the activity of the hydrogels was subsequently assessed. The dual-antibiotic PEGT_35_-GsCPX hydrogel was selected to evaluate its inhibitory and bactericidal effects against both bacterial strains. Among the three hydrogels studied, PEGT_35_-GsCPX displayed the most distinct release behavior for both drugs, exhibiting the most gradual Gs release profile, particularly relative to the least cross-linked formulation. Moreover, its results were more reproducible than those of PEGT_25_-GsCPX, for which some inconsistencies were observed due to the eventual breakage of the hydrogel during handling. For the *in vitro* biological assays, PEGT_35_-GsCPX xerogels were obtained by lyophilization, cut into small pieces (0.1–4 mg), and subsequently incubated in culture medium. After 24 h, aliquots of the resulting supernatants (containing the released drugs) were collected and exposed to each bacterial strain. Table 2 summarizes the MIC and MBC values determined from these supernatants after 24 h of incubation of the materials in culture medium (the bactericidal activity at different concentrations is shown in Figure S9, Supporting Information). The activity of the system is therefore presented as a function of the xerogel-to-medium mass:volume ratio (μg:mL, Table 2, Xerogel), and the results are compared with the released concentrations of each antibiotic after 24 h, according to the cumulative release studies shown in Figure 4c (Table 2, Released antibiotics, see).

For MRSA, ratios of 135 and 200 of PEGT_35_-GsCPX:culture medium ratio (μg:mL) were required to inhibit and eradicate bacterial growth, respectively. According to the drug release studies, the corresponding concentrations of antibiotics released and therefore needed for growth inhibition were 0.78 and 5.7 μg/mL for Gs and CPX, respectively. As can be compared in Table 2, these concentrations are markedly lower than those required for the free antibiotics alone (1 and 16 μg/mL for Gs and CPX, respectively), and are comparable to those required for the free drugs in combination (0.5 and 8 μg/mL). This observation highlights the preservation of the additive effect of both drugs previously demonstrated for the free antibiotics, even after their release. Furthermore, while CPX alone failed to eradicate MRSA even at concentrations above 356 μg/mL, the combination system achieved complete eradication with only 1.15 and 8.5 μg/mL of released Gs and CPX, respectively.

For *P. aeruginosa*, markedly lower amount of the material was needed, with inhibitory and bactericidal effects determined for a ratio range between 2.5 and 5 μg:mL, and 20 μg:mL of PEGT_35_-GsCPX:culture medium, respectively. Since the MIC values of both free antibiotics were similar (Table 2, Free antibiotics) and the amount of Gs released after 24 h was relatively low compared to CPX (Table 2, Released antibiotics), the concentration of released CPX required for growth inhibition was comparable to that of the free antibiotic alone (0.1 μg/mL). However, the Gs and CPX concentrations needed to eradicate the Gram-negative strain after 24 h of release (0.11 and 0.85 μg/mL of Gs and CPX, respectively) were lower than those required for the free drugs alone (4 and 1 μg/mL for Gs and CPX, respectively). Notably, the supernatants from the unloaded-antibiotic xerogel PEGT_35_ showed no bactericidal activity at the MIC and MBC values of the material for both bacterial strains, displaying bacterial counts similar to those of the control samples (Figure S9, Supporting Information).

Overall, these results highlight, first, that the antibiotics do not undergo any inactivating chemical modification during their incorporation in the hydrogel and release, since the concentrations required to inhibit bacterial growth are equal to or lower than those reported for the free antibiotics. Moreover, the dose reduction achieved by combining both antibiotics within the same drug-delivery system improved antibacterial efficacy over the individual drugs, thereby lowering the likelihood of infection persistence and the development of resistant strains.

Wound dressings should not have any negative impact on the regenerative cells that are involved during the wound healing process. To evaluate the cytocompatibility of PEGT_35_-GsCPX, human dermal fibroblasts and J774 macrophages were selected for the analysis, since they are involved in the initial healing and immune responses, respectively.^61^ Figure 5 shows the percentages of viable cells after being exposed for 24 h to the supernatant released from the coloaded PEGT_35_-GsCPX and unloaded PEGT_35_ xerogels (dashed lines, studied only at the MBC for each stain), compared to the control samples (not treated cells, assigned with 100% viability).

**Figure 5:**
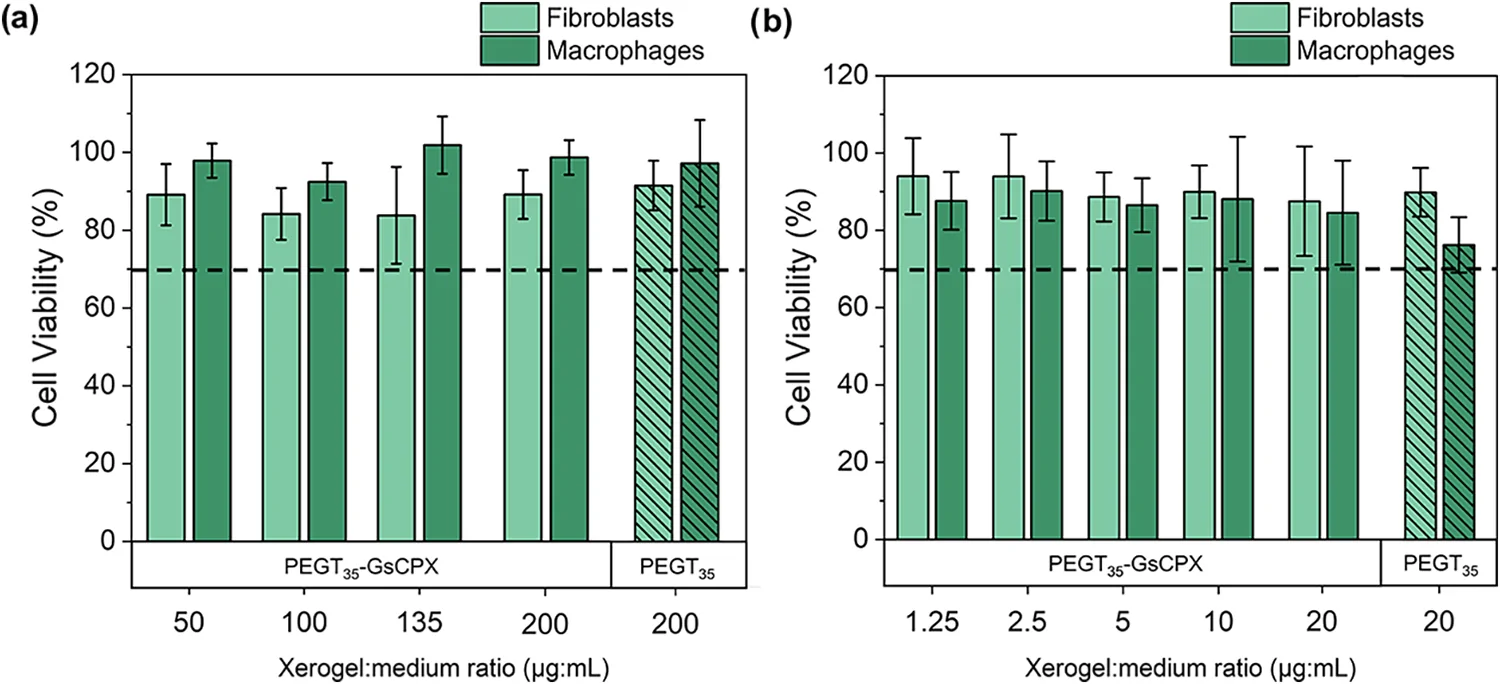
Cell viability (%) in fibroblast and macrophage cultures exposed to the supernatant released after 24 h from PEGT_35_-GsCPX and unloaded PEGT_35_ xerogels at the xerogel-to-medium ratios used for (a) MRSA studies and (b) *P. aeruginosa* studies. The dotted line indicates the 70% viability threshold in accordance with ISO 10993–5. Control samples were considered as 100% viability. Three replicates of each concentration were tested in triplicate (*n* = 9).

Tests were performed at the concentrations used for MRSA (Figure 5a) and *P. aeruginosa* (Figure 5b) MIC and MBC studies. In all cases, the cell viability of both cell lines remained above 70% at the concentrations tested, which complies with the ISO 10993–5:2009 standard, stating that a medical device is considered cytocompatible when cell viability stays above 70%.^62^ Our results are consistent with previous studies reporting the cytocompatibility of β-aminoacrylate-based hydrogels in NIH-3T3 and L929 fibroblasts,^27,28,63^ as well as β-thioacrylate hydrogels in human mesenchymal stem cells and 3T3 fibroblasts.^31,64^ Gs and CPX have been also widely shown to be noncytotoxic in various cell lines at the concentration ranges used in our assays.^13,65^ In conclusion, supernatants from PEGT_35_-GsCPX hydrogel were considered noncytotoxic for the cell lines studied at the doses at which bacteria were eradicated. These findings are highly relevant for the intended application in the treatment of infected topical wounds, where maintaining the integrity of patient́s cells is essential to ensure proper tissue regeneration.

## 4 Conclusions

Dual-antibiotic hydrogels incorporating Gs through amino-yne covalent conjugation and CPX via physical loading have been fabricated for their potential application as combinatorial wound dressings. The cross-linking strategy was based on the reaction of PEGalk with both Gs and DTT through amino-yne and thiol-yne chemistries, respectively. Increasing the molar percentage of thiol groups (15, 25, and 35% mol) relative to alkynoates resulted in a higher cross-linking degree, leading to faster gelation times and reduced swelling capacity. These effects were attributed to the formation of denser and highly cross-linked networks, as confirmed by the increased gel fraction.

As a result, CPX drug loading and encapsulation efficiency were able to be modulated, as well as CPX and Gs release kinetics, with PEGT_35_-GsCPX hydrogel exhibiting the most reproducible and controlled drug delivery behavior. Notably, the presence of both antibiotics within the hydrogel, physically and covalently bound, provided an initial burst release of CPX followed by a sustained release of Gs over time at physiological pH, with faster release kinetics of both drugs under mildly acidic conditions (pH = 5.0), as consequence of the cleavage of β-aminoacrylate linkages connecting Gs to the hydrogel network. This dual-release behavior not only highlights the utility of β-aminoacrylate linkages for controlling drug release rates but also offers a strategic advantage in preventing bacterial colonization: CPX could allow for the immediate elimination of invading bacteria after wounding, while Gs maintains antimicrobial activity over time, thereby minimizing the risk of reinfection.

Exudates obtained from the antibiotic-loaded hydrogels were able to inhibit bacterial growth and eradicate both MRSA and *P. aeruginosa* in the conducted *in vitro* experiments. Moreover, the additive effect previously demonstrated between Gs and CPX as free drugs was preserved after their release from the material, highlighting the potential of this system as a combinatorial therapeutic platform. Finally, the hydrogel supernatants were shown to be noncytotoxic toward human dermal fibroblasts and J774 macrophages at antimicrobial concentrations. Overall, these results support the suitability of the developed hydrogels as safe and effective dual-antibiotic wound dressings.

## Supplementary Material



## Notes

Details regarding the preparation of PEGT_*x*_-Gs, PEGT_*x*_, and %PEG-Gs hydrogels; rheological properties of PEGT_35_-GsCPX swollen hydrogels, mean pore size of PEGT_*x*_-Gs and PEGT_*x*_-GsCPX xerogels; calibration curves for Gs and CPX quantification; hydrogel swelling and degradation studies; mathematical fitting of Gs and CPX release profiles; and bactericidal activity at different concentrations of free Gs and CPX, as well as after their release from the hydrogels (PDF)
