# Supplementary material for: Dual Release of Antibiotics with Additive Effect from Cleavable Hydrogels Based on Amino/Thiol–Yne Click Chemistry
Source: Biomacromolecules. 2026 Aug 17;27(9):6175–89. doi: 10.1021/acs.biomac.6c01230 (PMC13580029; doi:10.1021/acs.biomac.6c01230)
Supplement: Supplementary file 1 [file bm6c01230_si_001.pdf]

Supporting Information

Dual Release of Antibiotics with Additive Effect from Cleavable Hydrogels Based on Amino/Thiol-Yne Click Chemistry

Sara Bescós-Ramo[1,2], Cristina Yus[1,3,4], Manuel Arruebo[1,3,4], Milagros Piñol[1,2], Luis Oriol[1,2*]*

[1]Instituto de Nanociencia y Materiales de Aragón (INMA), CSIC-Universidad de Zaragoza, Zaragoza 50009, Spain.

[2]Departamento de Química Orgánica, Facultad de Ciencias, Universidad de Zaragoza, 50009 Zaragoza, Spain.

[3]Department of Chemical Engineering and Environmental Technology, Universidad de Zaragoza, 50018, Zaragoza, Spain

[4]Aragon Health Research Institute (IIS Aragón), 50009, Zaragoza, Spain

1. **Synthesis and Characterization of Hydrogels**

Table S1. Mass, volume and concentration of reagents used for the preparation of $PEGT_x$-Gs hydrogels.

Sample	PEGalk (mg)	PB for PEGalk (µL)	Gs solution 45.1 mg/mL (µL)	DTT solution (mg/mL / µL)
$PEGT_{15}$-Gs	20	40	20	4.8 / 20
$PEGT_{25}$-Gs	20	40	20	8.0 / 20
$PEGT_{35}$-Gs	20	40	20	11.1 / 20

$PEGT_x$ hydrogel preparation. Hydrogels were prepared in 1.5 mL plastic vials (8 × 35 mm) with a final mass of 100 mg (PEGalk + solvent). First, 20 mg of PEGalk were weighed with into the plastic vial and dissolved in 60 µL of PB by orbital stirring for 4 h at room temperature. DTT solution in PB was prepared so that 1:0.15/0.25/0.35 molar ratio of alkynoate:thiol groups were reached after adding 20 µL of the solution. After 10 s of vortex mixing, hydrogels were allowed to set at a controlled temperature of 25 ºC for 18 h. Only those containing 1:0.35 molar ratio of alkynoate:thiol groups formed self-standing hydrogels, named as $PEGT_{35}$.

Table S2. Mass, volume and concentration of reagents used for the preparation of $PEGT_x$ hydrogels, and gelation time determined by inverted vial test.

Sample	PEGalk (mg)	Volume of PB for PEGalk (µL)	Concentration of DTT solution (mg/mL)	Volume of DTT solution (µL)	Gelation time (s)
$PEGT_{15}$	20	60	4.8	20	No gel
$PEGT_{25}$	20	60	8.0	20	No gel
$PEGT_{35}$	20	60	11.1	20	300 ± 5

%PEG-Gs hydrogel preparation. Hydrogels were prepared in 1.5 mL plastic vials (8 × 35 mm) with a final mass of 100 mg (PEGalk + solvent). First, appropriate amount of PEGalk was weighted in the glass vial to achieve polymer concentrations of 5, 10, 20 or

30 wt .% in the final mass. Then, it was dissolved in 50 % of final volume of PB by orbital stirring for 4 h at room temperature. Gs solution in PB was prepared so that 1:1 stoichiometric ratio of amino:alkynoate groups was reached after adding the amine in 50 % of final volume of PB. After 10 s of vortex mixing, hydrogels were allowed to set at a controlled temperature of 25 ºC for 18 h.

Table S3. Mass or volume of reagents and solvent for the preparation of %PEG-Gs hydrogels (being % the polymer concentration), and gelation time determined by inverted vial test.

Sample	PEGalk (mg)	Volume of PB for PEGalk (µL)	Concentration of Gs solution (mg/mL)	Volume of Gs solution (µL)	Gelation time
5PEG-Gs	5	47.5	4.8	47.5	No gel
10PEG-Gs	10	45	10	45	No gel
20PEG-Gs	20	40	22.6	40	Overnight
30PEG-Gs	30	35	38.7	35	Overnight

PEGT$_x$-GsCPX preparation.

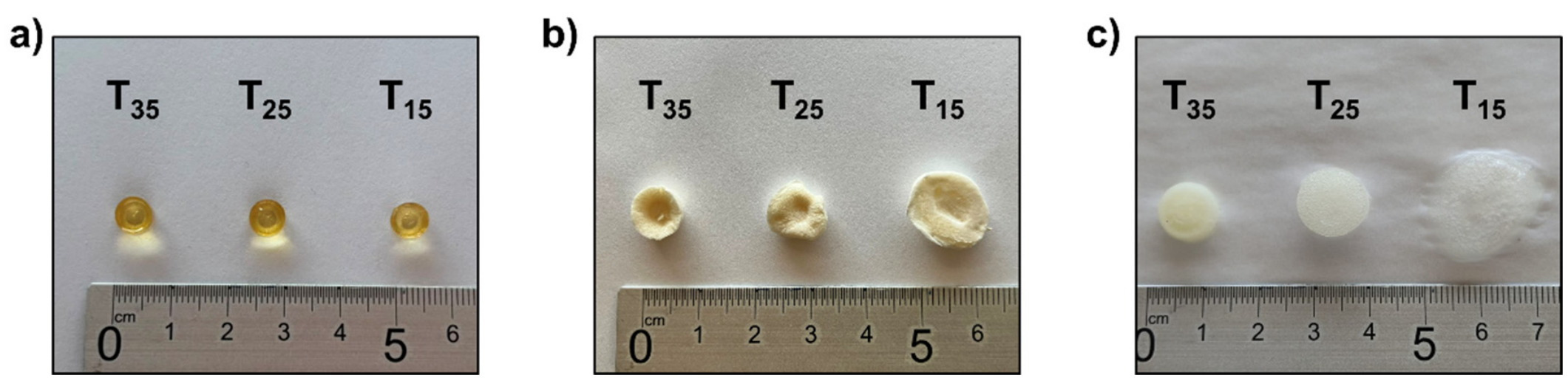

Figure S1. Images of PEGT$_x$-GsCPX hydrogels: (a) 18 h after preparation; (b) xerogels after washing with PB and lyophilization; and (c) hydrogels after CPX loading.

Attenuated Total Reflection (ATR) infrared spectroscopy.

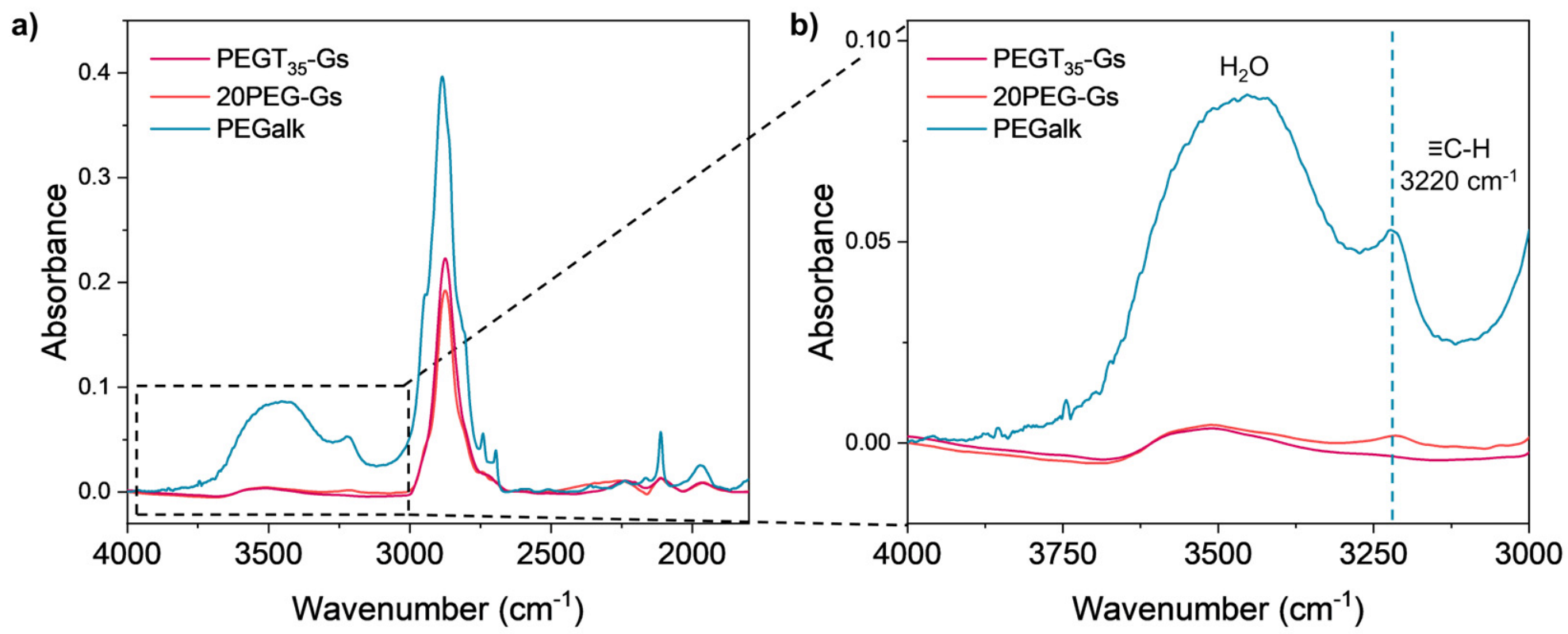

Figure S2. ATR FTIR absorbance spectra of PEGalk, 20PEG-Gs xerogel, and $PEGT_{35}$-Gs xerogel. (a) Spectra in the 4000-1800 cm^{-1} region showing the characteristic alkyne stretching vibrations. (b) Enlarged view of the 4000-3000 cm^{-1} region highlighting the ≡C-H stretching band (~3220 cm^{-1}), which was used to assess residual alkyne groups, as the C≡C stretching region is affected by atmospheric correction artifacts. Compared with PEGalk, the ≡C-H stretching band is markedly reduced in 20PEG-Gs, although residual alkyne groups remain detectable, which are no longer observed in $PEGT_{35}$-Gs after DTT-mediated cross-linking.

Rheological characterization of the swollen hydrogels.

Table S4. Storage (G′) and loss (G″) moduli of PB-swollen $PEGT_{35}$-Gs hydrogels and CPX-loaded $PEGT_{35}$-GsCPX hydrogels, measured at 37 °C using a constant frequency of 0.1 Hz and a constant strain amplitude of 0.05. Data are presented as the mean ± SD of three independent samples (n = 3).

Sample	G’ (kPa)	G’’ (kPa)
**$PEGT_{35}$-Gs** (immersed in pH 7.4 buffer, PB)	15.3 ± 0.7	1.7 ± 0.4
**$PEGT_{35}$-GsCPX** (immersed in CPX solution)	13.0 ± 2.1	0.8 ± 0.2

Morphological characterization: pore size.

Table S5. Mean pore size (µm) of 20 % wt. $PEGT_x$-Gs and $PEGT_x$-GsCPX xerogels measured by FESEM.

Sample	Pore size (µm)
$PEGT_{15}$-Gs	105 ± 22
$PEGT_{15}$-GsCPX	109 ± 18
$PEGT_{25}$-Gs	95 ± 22
$PEGT_{25}$-GsCPX	98 ± 27
$PEGT_{35}$-Gs	94 ± 33
$PEGT_{35}$-GsCPX	96 ± 27

2. Antibiotics Determination and Release Studies

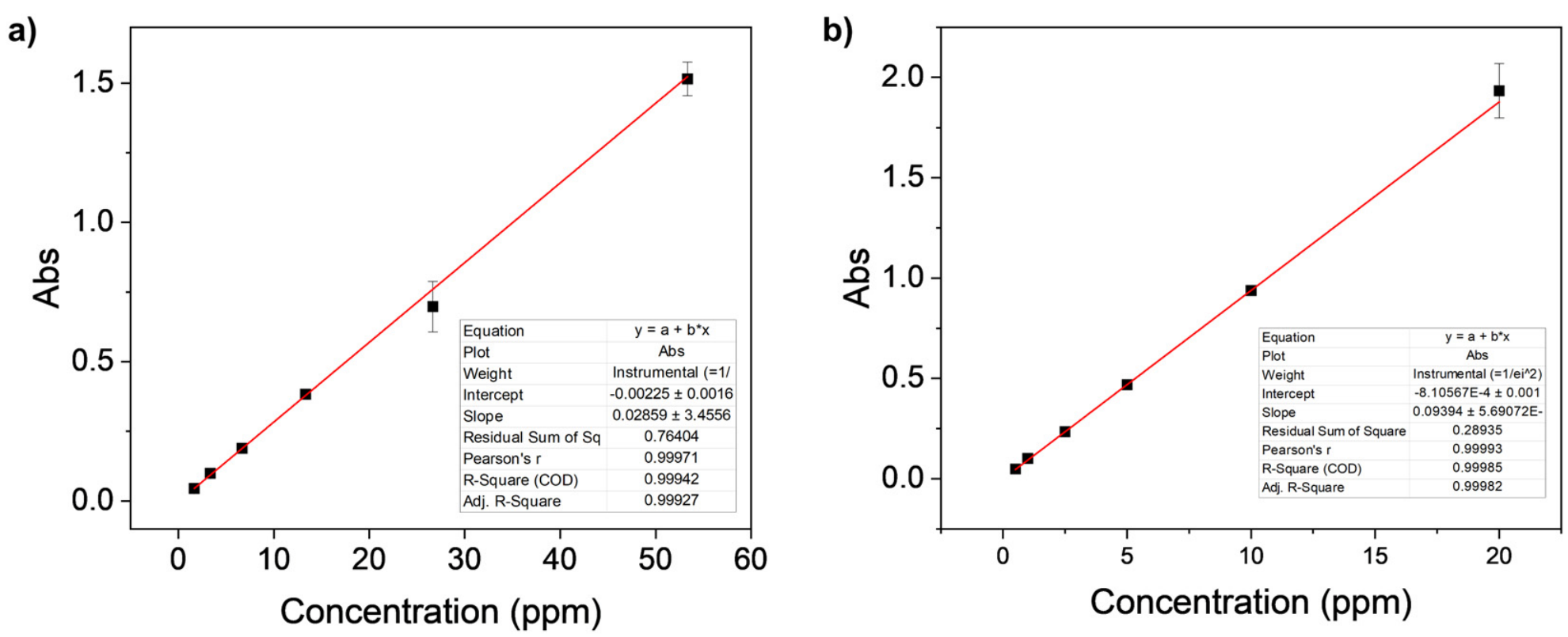

Figure S3. Calibration curves for (a) Gs and (b) CPX, showing absorbance as a function of concentration at 333 and 271 nm, respectively, with linear fits ($R^2 > 0.999$).

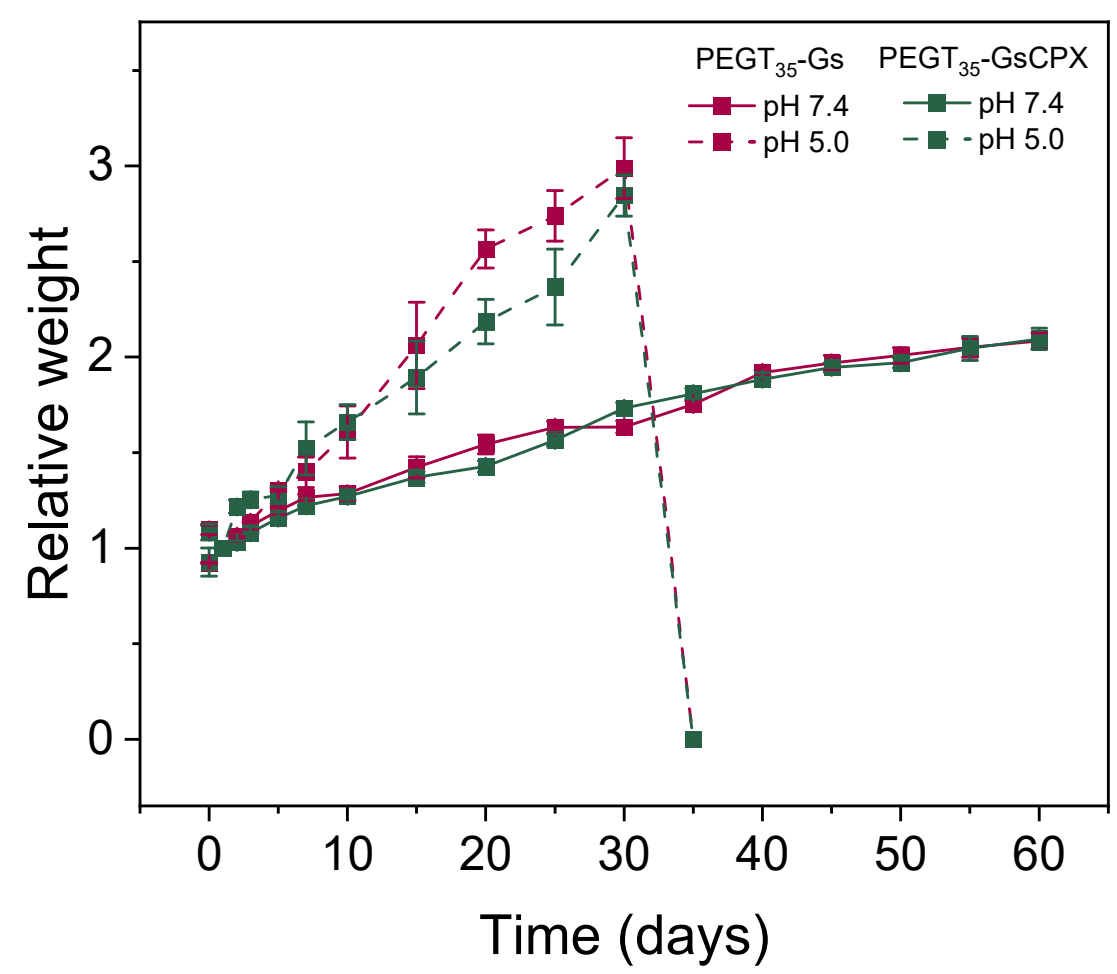

Figure S4. Relative weight of PEGT35-Gs (pink lines) and PEGT35-GsCPX hydrogels (green lines) over 60 days at pH=7.4 (solid lines) and pH=5.0 (dashed lines); the abrupt decrease observed on day 35 is attributed to hydrogel solubilization between days 30 and 35.

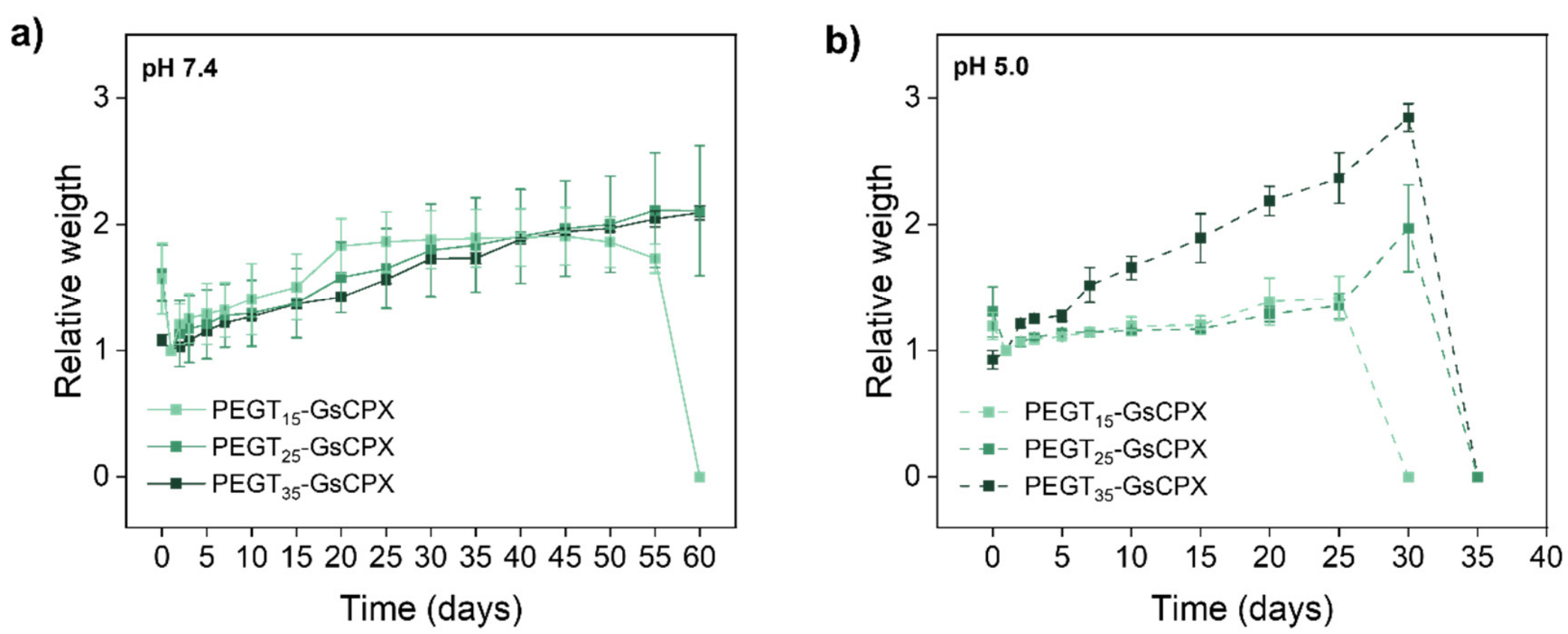

Figure S5. Relative weight of PEGT15-GsCPX, PEGT25-GsCPX and PEGT35-GsCPX hydrogels at a) pH=7.4 over 60 days and b) pH=5.0 over 35 days. As explained in the experimental section, the reference weight was selected as that of the hydrogel after 24 h in the chosen buffer (day 1), since a noticeable change in swelling was observed when the material was transferred from the CPX solution used in the swelling-diffusion method (day 0) to the buffer employed for the degradation studies. The abrupt decrease observed was attributed to hydrogel solubilization.

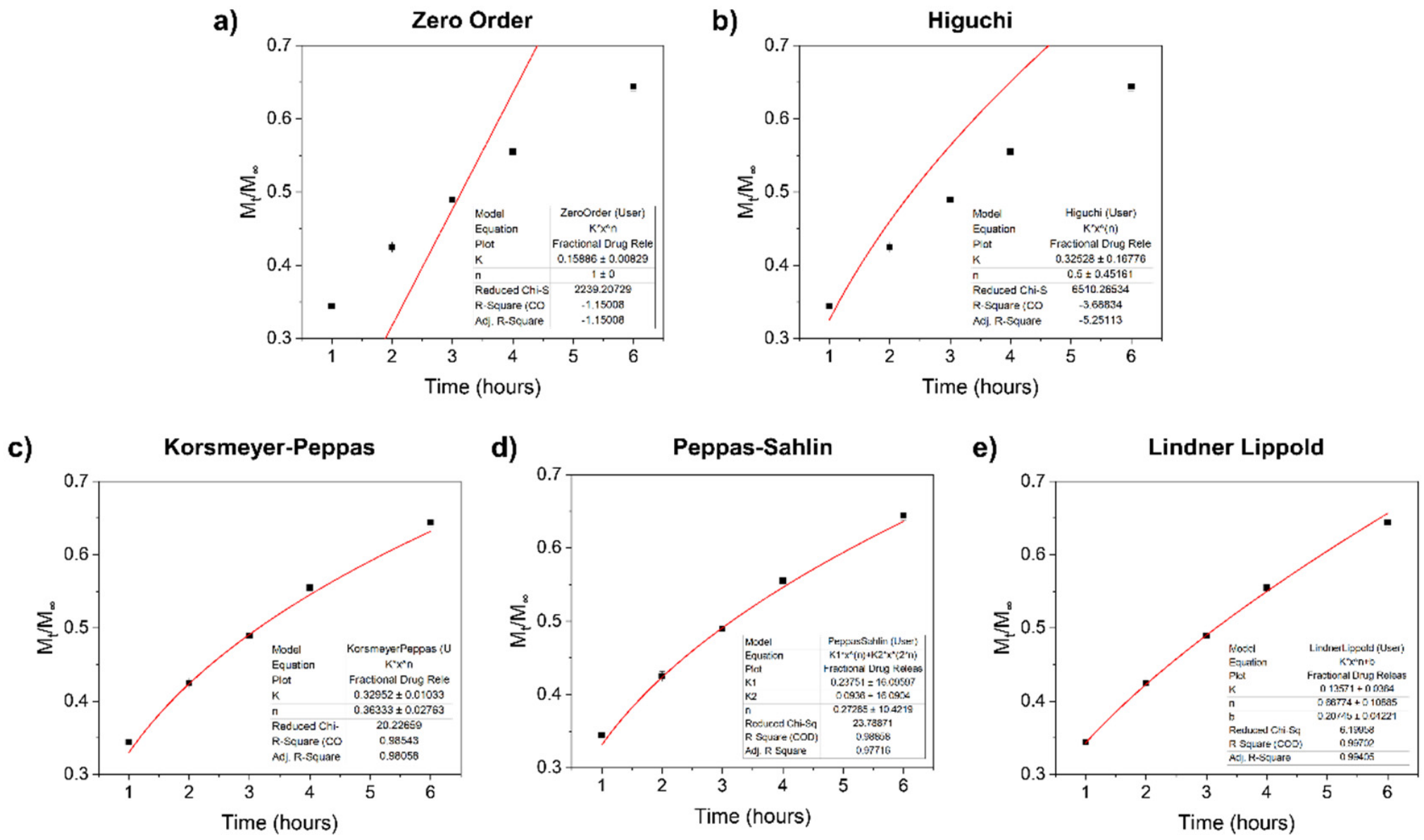

Figure S6. Mathematical fitting of CPX release kinetics at pH=7.4 from PEGT35-GsCPX hydrogels using a) first-order, b) Higuchi, c) Korsmeyer-Peppas, d) Peppas-Sahlin, and e) Lindner-Lippold models.

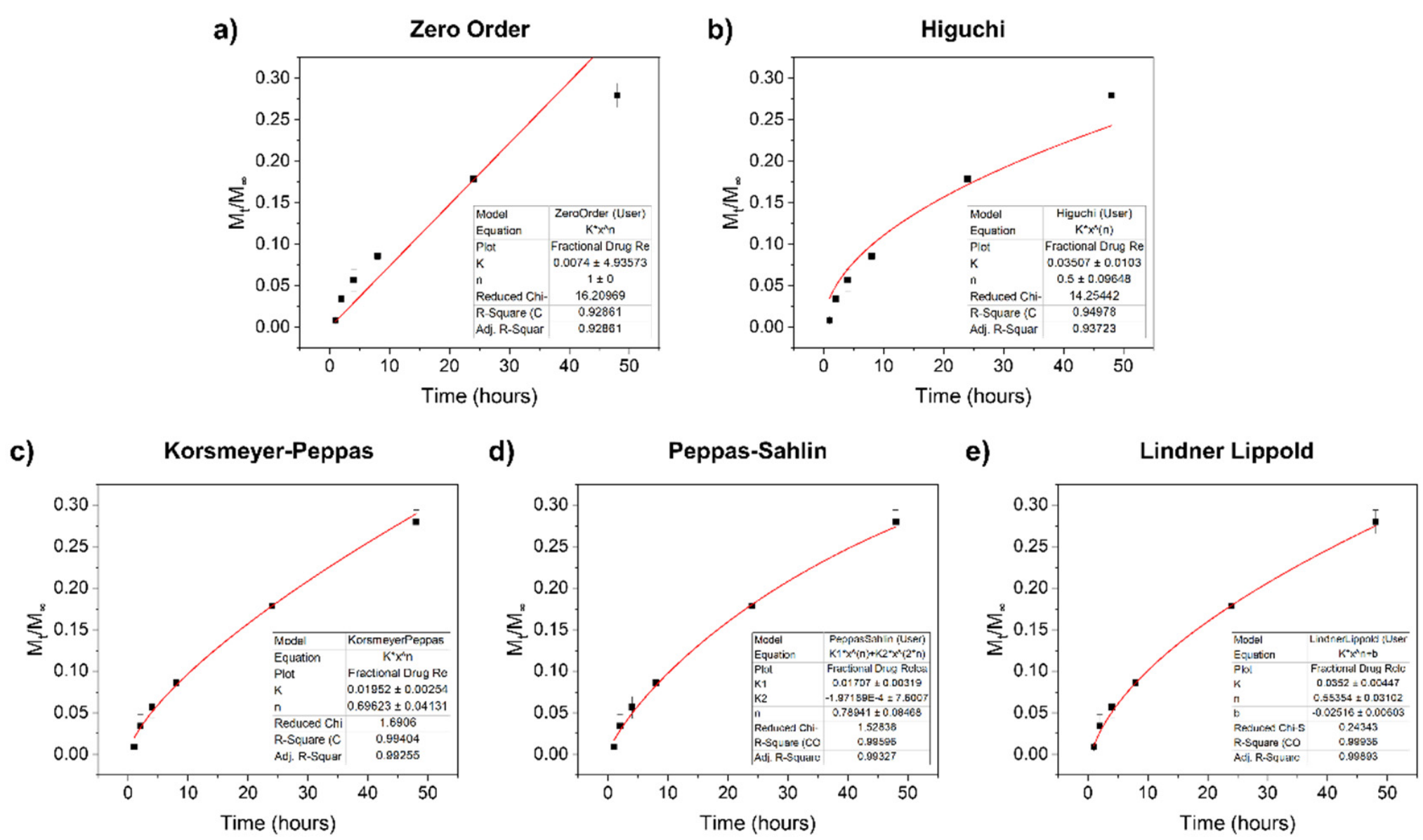

Figure S7. Mathematical fitting of Gs release kinetics at pH=7.4 from PEGT35-Gs hydrogels using a) first-order, b) Higuchi, c) Korsmeyer-Peppas, d) Peppas-Sahlin, and e) Lindner-Lippold models.

3. *In vitro* biological evaluation

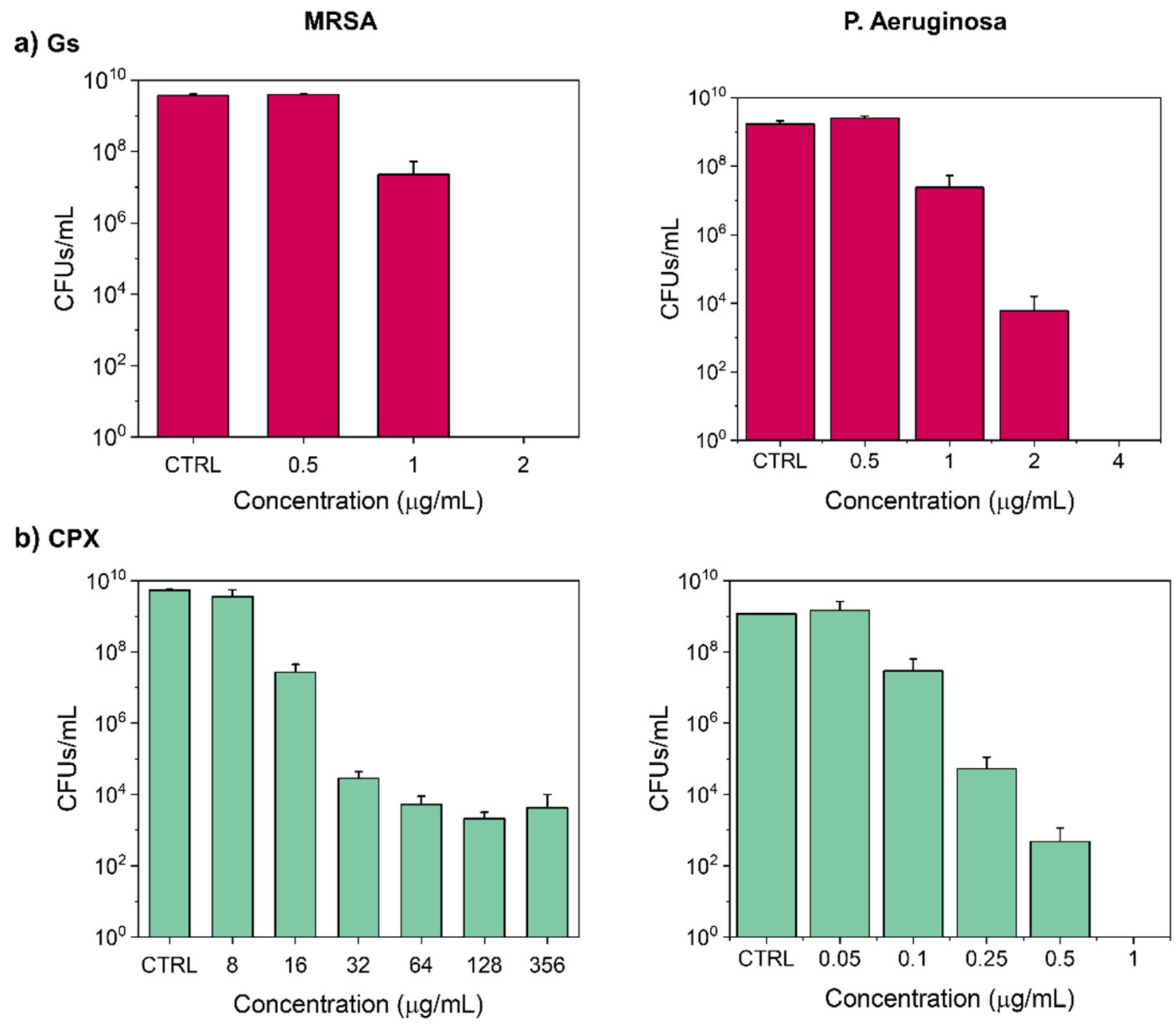

Figure S8. Bactericidal activity of (a) Gs and (b) CPX against MRSA (left) and *P. aeruginosa* (right). Data are presented as mean ± SD from three independent experiments performed in triplicate (n = 9).

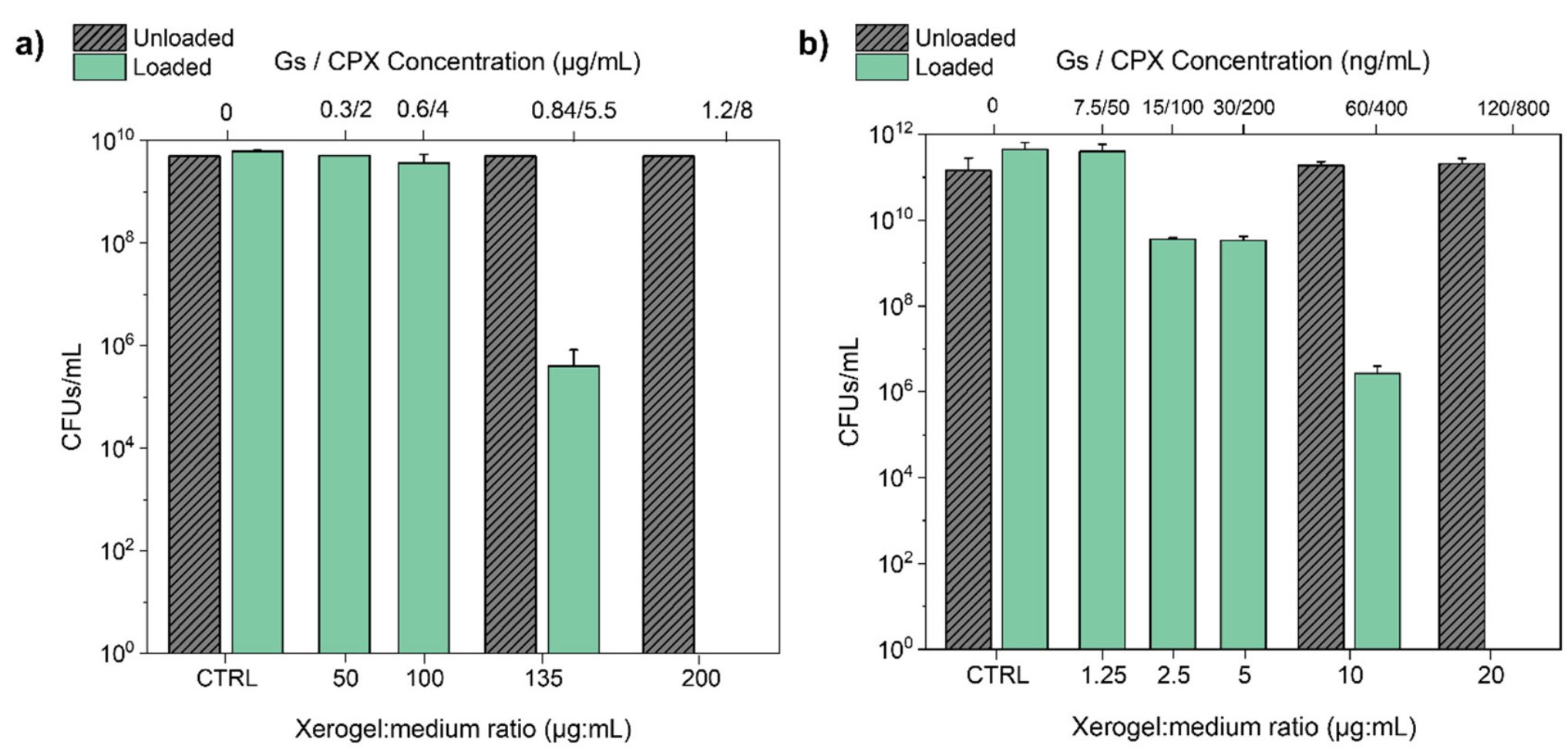

Figure S9. Bactericidal activity of the supernatant released after 24 h from $PEGT_{35}$-GsCPX (green) and unloaded $PEGT_{35}$ (gray) against (a) MRSA and (b) *P. aeruginosa*. The lower X-axis refers to the $PEGT_{35}$-GsCPX xerogel-to-medium mass:volume ratio (μg:mL), whereas the upper X-axis represents the released concentrations of each antibiotic (Gs/CPX) after 24 h according to the antibiotic release studies from the hydrogels. Data are presented as mean ± SD from three independent experiments performed in triplicate (n = 9).

Example calculation of the released antibiotic concentrations. The concentration of each antibiotic available in the culture medium for *in vitro* studies was estimated from the xerogel-to-medium ratio, the drug loading of the hydrogel, and the cumulative release after 24 h. As an example, the calculation corresponding to the MIC against MRSA (135 µg/mL xerogel-to-medium ratio) is shown below. Since the antibacterial assays were performed in 2 mL of tryptic soy broth (TSB), a total of 0.27 mg of $PEGT_{35}$-GsCPX xerogel was incubated. Considering the drug loading values of the hydrogels (3.2 wt% Gs and 4.6 wt% CPX), the amount of each antibiotic contained in 0.27 mg of xerogel was calculated as:

- Gs: 0.27 mg × 0.032 = 8.64 µg
- CPX: 0.27 mg × 0.046 = 12.42 µg

Based on the cumulative release after 24 h (18 % for Gs and 92 % for CPX), the amounts of antibiotics released into the medium were:

- Released Gs: 8.64 × 0.18 = 1.56 µg
- Released CPX: 12.42 × 0.92 = 11.43 µg

Finally, since the released antibiotics were diluted in 2 mL of TSB, the concentrations available to inhibit bacterial growth were:

- Gs: 1.56 µg / 2 mL = 0.78 µg/mL
- CPX: 11.43 µg / 2 mL = 5.71 µg/mL

The same calculation procedure was applied to all the xerogel-to-medium ratios evaluated in this study.
